# Conventional and Chemically Programmed Asymmetric Bispecific Antibodies Targeting Folate Receptor 1

**DOI:** 10.3389/fimmu.2019.01994

**Published:** 2019-08-21

**Authors:** Junpeng Qi, David Hymel, Christopher G. Nelson, Terrence R. Burke, Christoph Rader

**Affiliations:** ^1^Department of Immunology and Microbiology, The Scripps Research Institute, Jupiter, FL, United States; ^2^Chemical Biology Laboratory, Center for Cancer Research, National Cancer Institute, National Institutes of Health, Frederick, MD, United States

**Keywords:** bispecific antibodies, catalytic antibodies, folate, FOLR1, CD3, ovarian cancer

## Abstract

T-cell engaging bispecific antibodies (biAbs) can mediate potent and specific tumor cell eradication in liquid cancers. Substantial effort has been invested in expanding this concept to solid cancers. To explore their utility in the treatment of ovarian cancer, we built a set of asymmetric biAbs in IgG1-like format that bind CD3 on T cells with a conventional scFv arm and folate receptor 1 (FOLR1) on ovarian cancer cells with a conventional or a chemically programmed Fab arm. For avidity engineering, we also built an asymmetric biAb format with a tandem Fab arm. We show that both conventional and chemically programmed CD3 × FOLR1 biAbs exert specific *in vitro* and *in vivo* cytotoxicity toward FOLR1-expressing ovarian cancer cells by recruiting and activating T cells. While the conventional T-cell engaging biAb was curative in an aggressive mouse model of human ovarian cancer, the potency of the chemically programmed biAb was significantly boosted by avidity engineering. Both conventional and chemically programmed CD3 × FOLR1 biAbs warrant further investigation for ovarian cancer immunotherapy.

## Introduction

As one of the promising next-generation cancer immunotherapeutics, T-cell engaging bispecific antibodies (biAbs) mediate potent and selective cytotoxicity by targeting tumor cell surface receptors with one arm and by recruiting and activating T cells with the other arm (1, 2). The 2014 FDA approval of CD3 × CD19 biAb blinatumomab (Blincyto^®^) for the treatment of adults and children with refractory or relapsed pre-B cell acute lymphoblastic leukemia (pre-B-ALL) followed by its 2018 FDA approval for the treatment of adults and children with pre-B ALL in first or second complete remission with minimal residual disease, are milestones marking the therapeutic utility of T-cell engaging biAbs (3–5). At least 25 T-cell engaging biAbs are currently investigated in phase I and II clinical trials for the treatment of liquid and solid malignancies (2).

Conventional biAbs combine two different monoclonal antibodies (mAbs) or antibody fragments for binding to T cells and tumor cells. For example, blinatumomab is composed of an anti-CD3 single-chain Fv (scFv) adjoined to an anti-CD19 scFv. These antibody fragments, typically scFv, or Fab, can be connected through an Fc module for prolonged circulatory half-life (6). The distinguishing feature of chemically programmed biAbs is the use of a natural or synthetic small molecule rather than an antibody or antibody fragment for tumor cell binding and site-specific covalent conjugation to a T-cell engaging antibody or antibody fragment (7–10). The molecularly defined assembly of a variable chemical component that targets a tumor cell surface receptor and an invariable biological component that recruits and activates T cells affords a highly versatile and economically attractive composition (11). The concept of chemically programmed biAbs builds on an anticipated wealth of synthetic small molecules derived from chemical libraries or from structure-based design campaigns to bind tumor cell surface receptors with high specificity and affinity.

In the present study, we report a new set of chemically programmed biAb formats that endow small molecules with the circulatory half-life and avidity of mAbs. The chemically programmable module is a humanized mAb, h38C2, in Fab format (12). It is based on a family of catalytic antibodies which were raised by reactive immunization of mice with a 1,3-diketone hapten and which harbor a uniquely reactive lysine residue at the bottom of an 11-Å deep hydrophobic cleft in the antibody combining site (13–15). The reactive lysine forms a reversible covalent enamine adduct with 1,3-diketone hapten derivatives. As such, it has been used as chemically programmable module of chemically programmed antibodies (16). Subsequent adaptations of this concept involved β-lactam hapten derivatives for irreversible covalent amide adduct formation which ultimately led to phase I and II clinical trials of chemically programmed antibodies enrolling hundreds of cancer and type 2 diabetes patients (11). Our new set of chemically programmed biAb formats builds on this track record of h38C2 as a chemically programmable and clinically translatable module.

To compare conventional and chemically programmed biAbs side-by-side, we chose folate receptor 1 (FOLR1; also known as folate receptor α) as a tumor cell surface receptor. FOLR1 is a glycosylphosphatidylinositol (GPI)-anchored glycoprotein. It binds folate (vitamin B9), a natural small molecule, with nanomolar affinity and mediates its transmembrane transport via receptor-mediated endocytosis (17). Its overexpression in various solid malignancies including ovarian and lung cancer makes FOLR1 an attractive target for both small molecule- and antibody-based diagnostic and therapeutic reagents (18, 19). This setting, along with a pressing public health need for new cancer immunotherapy strategies in ovarian and lung cancer, makes FOLR1 an ideal target for developing and comparing conventional and chemically programmed biAbs that recruit and activate T cells.

## Materials and Methods

### Small Molecules

Methodol (20) and β-lactam-biotin-folate **1b** (10) were synthesized as described. The syntheses of β-lactam-biotin-folate **1a**, β-lactam-biotin-(folate)_2_ **2**, and β-lactam-biotin-LLP2A **3** are described in section Supplementary Materials and Methods.

### Cell Lines and Primary Cells

Human T-cell line Jurkat and human mantle cell lymphoma cell line JeKo-1 were obtained from the American Type Culture Collection (ATCC) and cultured in RPMI-1640 medium supplemented with L-glutamine, 100 U/mL Penicillin-Streptomycin, and 10% (v/v) fetal calf serum (FCS; all from Thermo Fisher Scientific). NCI-60 panel human ovarian cancer cell lines IGROV-1 and SKOV-3 were obtained from The Scripps Research Institute's Cell-based Screening Core and cultured in folate-deficient RPMI 1640 medium supplemented with L-glutamine, 100 U/mL Penicillin-Streptomycin, and 10% (v/v) FCS. IGROV-1 cells stably transfected with firefly luciferase (IGROV-1/ffluc) were previously described (10). Expi293F cells (Thermo Fisher Scientific) were grown in Expi293 Expression Medium (Thermo Fisher Scientific) supplemented with 100 U/mL Penicillin-Streptomycin. PBMC were isolated from healthy donor buffy coats using Lymphoprep (Axis-Shield) and cultured in X-VIVO 20 medium (Lonza) with 5% (v/v) off-the-clot human serum AB (Gemini Bio-Products) and 100 U/mL IL-2 (Cell Sciences). Primary T cells were expanded from PBMC as previously described (21) using Dynabeads Human T-Activator CD3/CD28 (Thermo Fisher Scientific). All primary T cells in this study were expanded from the same healthy donor PBMC.

### Cloning, Expression, and Purification of Asymmetric biAbs

All amino acid sequences have been previously published or patented and are compiled in section Supplementary Materials and Methods. Heterodimeric biAbs with knobs-into-holes mutations in the Fc module (22) and mAb v9 in scFv format were cloned as previously described (21). The variable domain encoding cDNA sequences of mAbs Farletuzumab (Farl) and h38C2 were PCR-amplified from previously described plasmids (10, 23). A EPKSCD(G_4_S)_2_ linker encoding sequence was used to fuse two h38C2 Fab by overlap extension PCR. To express asymmetric biAbs, three plasmids encoding (i) a v9 scFv-hinge-C_H_2-C_H_3 (holes) polypeptide, (ii) a Farl, h38C2, or (h38C2)_2_ heavy chain (knobs) polypeptide, and (iii) a Farl or h38C2 light chain polypeptide were transiently co-transfected into Expi293F cells using the ExpiFectamine 293 Transfection Kit (Thermo Fisher Scientific) with an (i):(ii):(iii) ratio of 1:1:2. One week later, supernatants were collected followed by affinity chromatography using 1-mL Protein A HiTrap HP columns in conjunction with an ÄKTA FPLC instrument (both from GE Healthcare Life Sciences). Subsequent preparative and analytic size exclusion chromatography was performed with a Superdex 200 10/300 GL column (GE Healthcare Life Sciences) in conjunction with an ÄKTA FPLC instrument using PBS at a flow rate of 0.5 mL/min. The purity of the biAbs was confirmed by SDS-PAGE followed by Coomassie Blue staining, and their concentration was determined by measuring the absorbance at 280 nm. The negative control biAb 0 × (h38C2)_2_ was cloned, expressed, and purified as described above by replacing the v9 scFv-hinge-C_H_2-C_H_3 (holes) cassette with an empty hinge-C_H_2-C_H_3 (holes) cassette.

### Chemical Programming of biAbs

Purified biAbs at 10 μM were chemically programmed by incubation with 2 equivalents (20 μM) β-lactam-biotin-folate derivatives, incubated for 4 h at room temperature, and purified with a 50-kDa MWCO Amicon Ultrafiltration Unit (Millipore).

### Catalytic Activity Assay

Purified biAbs before and after chemical programming were diluted to 1 μM in PBS (pH 7.4) and dispensed in 98-μL aliquots into a 96-well plate in triplicate. Then, 2 μL of 10 mM methodol in ethanol was added and the fluorescence was assessed immediately using a SpectraMax M5 instrument (Molecular Devices) with SoftMax Pro software, the wavelength of excitation (λ ext) set to 330 nm, the wavelength of emission (λ em) set to 452 nm, and starting at 0 min using 5-min time points. The slopes were compared using linear regression analysis with GraphPad Prism 5 software.

### Flow Cytometry

Cells were stained with titrated conventional and chemically programmed biAbs using standard flow cytometry methodology. Secondary polyclonal antibodies (pAbs), APC or Alexa Fluor 647-conjugated goat anti-human or mouse IgG Fc-specific, were purchased from Jackson ImmunoResearch Laboratories. Alexa Fluor 647-conjugated mouse anti-human CD69 mAb was purchased from BioLegend. Mouse anti-human CD4 (OKT4) and CD8 (SK1) mAbs were purchased from BioLegend. For the cross-linking assay, target and effector cells were labeled with CellTrace CFSE and CellTrace Far Red (both from Thermo Fisher Scientific), respectively, according to the manufacturer's protocol. The labeled target and effector cells at a 1:5 ratio were then incubated with 200 nM biAbs in 100 μL final volume. Following incubation for 2 h at 37 °C, the cells were gently washed and analyzed by flow cytometry on a FACSCanto (BD Biosciences). Data were analyzed with WinMDI 2.9 software.

### *In vitro* Cytotoxicity Assay

Cytotoxicity was measured using CytoTox-Glo (Promega) following the manufacturer's protocol with minor modifications. Primary T cells expanded from healthy donor PBMC as described above were used as effector cells and IGROV-1, SKOV-3, or JeKo-1 cells were used as target cells. The cells were incubated at an effector-to-target (E:T) ratio of 10:1 in X-VIVO 20 Medium (Lonza) with 5% (v/v) off-the-clot human serum AB (Innovative Research). The target cells (2 × 10^4^) were first incubated with the biAbs prior to adding the effector cells (2 × 10^5^) in a final volume of 100 μL/well in a 96-well tissue culture plate. The plates were incubated for 16 h at 37 °C with biAb concentrations ranging from 0.08 to 500 nM. After centrifugation, 50 μL of the supernatant was transferred into a 96-well clear bottom white walled plate (Costar 3610; Corning) containing 25 μL/well CytoTox-Glo. After 15 min at room temperature, the plate was read using a SpectraMax M5 instrument with SoftMax Pro software. The same supernatants (diluted 10-fold) used for the CytoTox-Glo assay were also used to determine IFN-γ, IL-2, and TNF-α secretion with Human IFN-γ, IL-2, or TNF-α ELISA MAX™ Deluxe kits (BioLegend), respectively, following the manufacturer's protocols.

### Mouse Xenograft Studies

Twenty-five 6-weeks old NOD-scid-IL2Rγnull (NSG) mice (The Jackson Laboratory) were each given 1 × 10^6^ IGROV-1/ffluc intraperitoneally (i.p.) on day 0. On day 6, the animals were i.p. injected with 150 mg/kg D-luciferin (Biosynth) and divided into 5 groups of 5 animals each by average bioluminescence. On day 6, each mouse was i.p. injected with 1 × 10^7^ primary T cells expanded from healthy donor PBMC as described above, and 1 h later, with 12.5 μg v9 × Farl, 17.5 μg, or 52.5 μg folate-programmed v9 × (h38C2_**1b**)_2_, 52.5 μg unprogrammed v9 × (h38C2)_2_ or PBS alone. The mice received a total of 3 doses of expanded primary T cells every 8 days and a total of 6 doses of biAbs or PBS alone every 4 days. Every 3–5 days, tumor growth was monitored by bioluminescent imaging 5 min after i.p. injections with 150 mg/kg D-luciferin. For this, mice were anesthetized with isoflurane and imaged using an Xenogen IVIS Imaging System (Caliper) 6, 8, and 10 min after luciferin injection in small binning mode at an acquisition time of 10 s to 1 min to obtain unsaturated images. Luciferase activity was analyzed using Living Image software (Caliper) and the photon flux analyzed within regions of interest that encompassed the entire body of each individual mouse. The weight of the mice was measured every 3–4 days and euthanasia was performed when the mice gained more than 25% body weight due to increasing tumor burden and ascites volume. All procedures were approved by the Institutional Animal Care and Use Committee of The Scripps Research Institute and were performed according to the NIH Guide for the Care and Use of Laboratory Animals.

### Pharmacokinetic Study

Four female CD-1 mice (~25 g; Charles River Laboratories) were injected i.p. with v9 × h38C2_**1b**, v9 × (h38C2_**1b**)_2_, or v9 × Farl at 6 mg/kg. Using heparinized capillary tubes, blood was collected from the tail vein at 5 min, 30 min, 25 h, 49 h, 72 h, 97 h, 168 h, 240 h, and 336 h after injection. Plasma was obtained by centrifuging the samples at 2,000 × g for 5 min in a microcentrifuge and stored at −80°C until analysis. The concentrations of biAbs in the plasma samples were measured by flow cytometry. For this, 5 × 10^4^ IGROV-1 cells were incubated with the plasma samples for 1 h on ice followed by Alexa Fluor 647-conjugated goat anti-human IgG Fc-specific pAbs. The cells were gently washed and analyzed by flow cytometry on a FACSCanto (BD Biosciences). Using a standard curve based on the mean fluorescence intensity of known concentrations of biAbs, the concentration of the biAbs in the plasma samples was extrapolated from a four parameter logistic curve fit. Pharmacokinetic (PK) parameters were analyzed by using Phoenix WinNonlin PK/PD Modeling and Analysis software (Pharsight).

### Mouse Plasma Stability

The v9 × h38C2_**1b**, v9 × (h38C2_**1b**)_2_, and v9 × Farl biAbs were diluted to 200 μg/mL into Balb C mouse plasma (Innovative Research) and incubated at 37°C for 4 days. Control samples (0 days) were prepared by diluting the biAbs in mouse plasma immediately before flow cytometry. For this, 5 × 10^4^ IGROV-1 cells were stained with 40-fold dilutions of the incubated or control samples for 1 h on ice followed by Alexa Fluor 647-conjugated goat anti-human IgG Fc-specific pAbs. The cells were gently washed and analyzed by flow cytometry on a FACSCanto as described above.

### Statistical Analyses

Statistical analyses were performed using GraphPad Prism 5 software. The *in vitro* data shown in Figures 5B, 6, 7C, and Figure S6B were subjected to unpaired two-tailed *t*-test. Statistical analysis of survival (Figure 7D) was done by log-rank (Mantel-Cox) testing. Results with a *p*-value of *p* < 0.05 were considered significant.

## Results

### Asymmetric biAb Formats

**Figure 1:**
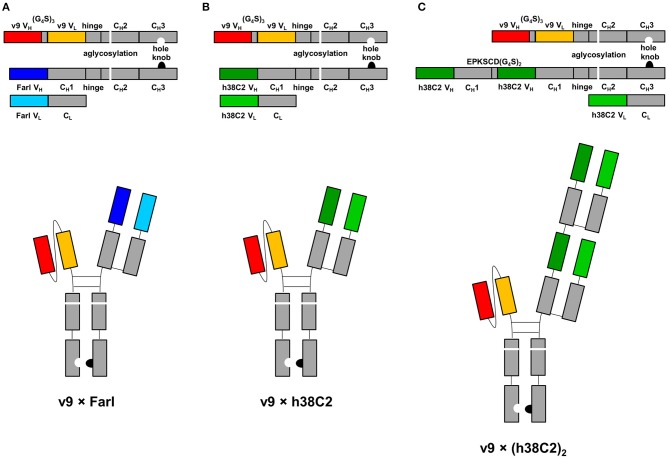
Design of asymmetric biAbs for conventional and chemically programmed cancer cell targeting. Schematic diagram of v9 × Farl **(A)**, v9 × h38C2 **(B)**, and v9 × (h38C2)_2_ **(C)**. All three asymmetric biAbs share a scFv module of v9, a humanized mouse anti-human CD3 mAb, and a Fc module of human IgG1 combining one mutation for aglycosylation in the C_H_2 domain (N297A) and two or four mutations in the C_H_3 knob (S354C and T366W) and C_H_3 hole (Y349C, T366S, L368A, and Y407V) domains, respectively, for heterodimerization. The Fab arm of the conventional biAb **(A)** is derived from humanized anti-human FOLR1 mAb farletuzumab (Farl). The single **(B)** or double **(C)** Fab arm of the chemically programmable biAbs is derived from humanized catalytic antibody 38C2 (h38C2).

The three asymmetric biAb formats employed in this study along with their expression cassettes are shown in Figure 1. All share (i) a human IgG1 Fc module with knobs-into-holes mutations for heterodimerization and aglycosylation mutation N297A in the second constant heavy chain domain (C_H_2), and (ii) a scFv module composed of the variable heavy (V_H_) and light (V_L_) chain domains of a humanized and affinity matured version, called v9, of mouse anti-human CD3 mAb UCHT1 (21). They differ with respect to their Fab arm. In the conventional biAb, the Fab arm is derived from the humanized anti-human FOLR1 mAb farletuzumab (Farl) (Figure 1A). The two chemically programmable biAbs bear a single (Figure 1B) or double Fab arm (Figure 1C) derived from humanized catalytic antibody 38C2 (h38C2). We will refer to these three biAbs as v9 × Farl, v9 × h38C2, and v9 × (h38C2)_2_, respectively. Each is composed of three different polypeptide chains encoded by three separate expression cassettes in the mammalian expression vector pCEP4. They include (i) a v9 scFv-hinge-C_H_2-C_H_3 (holes) polypeptide, (ii) a Farl, h38C2, or (h38C2)_2_ heavy chain (knobs) polypeptide, and (iii) a Farl or h38C2 light chain polypeptide. Note that the double Fab arm, which contains a EPKSCD(G_4_S)_2_ spacer between the two identical V_H_-C_H_1 units, uses two identical light chains. The three encoding plasmids of each biAb were combined by transient co-transfection into Expi293F human embryonic kidney cells. Following Protein A affinity chromatography that yielded ~50 mg/L v9 × Farl and v9 × h38C2, and ~30 mg/L v9 × (h38C2)_2_, non-reducing and reducing SDS-PAGE showed the expected bands and size exclusion chromatography (SEC) revealed <5% aggregates (Figures S1A–C). Sequential Protein A affinity chromatography and SEC were used to purify monomeric biAbs for all subsequent functional studies.

### Conventional and Chemically Programmed FOLR1 Binding

**Figure 2:**
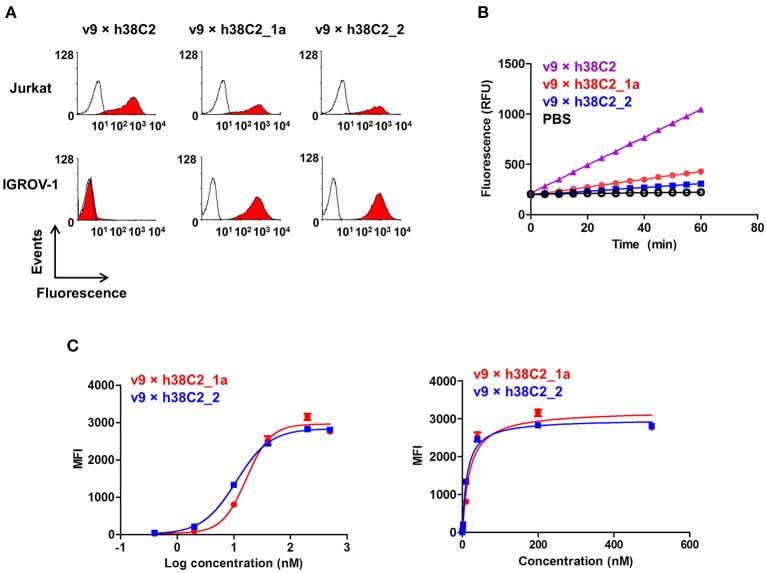
Cell surface binding and catalytic activity of chemically programmed FOLR1-targeting biAb with a single Fab arm. **(A)** Flow cytometry analysis of unprogrammed and chemically programmed biAbs binding to CD3+ FOLR1– human T-cell line Jurkat and FOLR1+ CD3– human ovarian cancer cell line IGROV-1 using 20 nM biAbs followed by Alexa Fluor 647-conjugated goat anti-human IgG-Fc pAbs. Compounds **1a** and **2** are monovalent and bivalent folate derivatives, respectively. **(B)** Catalytic retro-aldol activity of unprogrammed and chemically programmed h38C2-containing biAbs. The signal is reported in relative fluorescent units (RFU; mean ± SD of triplicates). PBS was used as negative control. **(C)** Titration curve of chemically programmed biAbs binding to IGROV-1 cells detected with Alexa Fluor 647-conjugated goat anti-human IgG pAbs (left panel). Saturation analysis of chemically programmed biAbs binding to IGROV-1 cells (right panel). Shown are mean ± SD values from independent triplicates.

**Figure 3:**
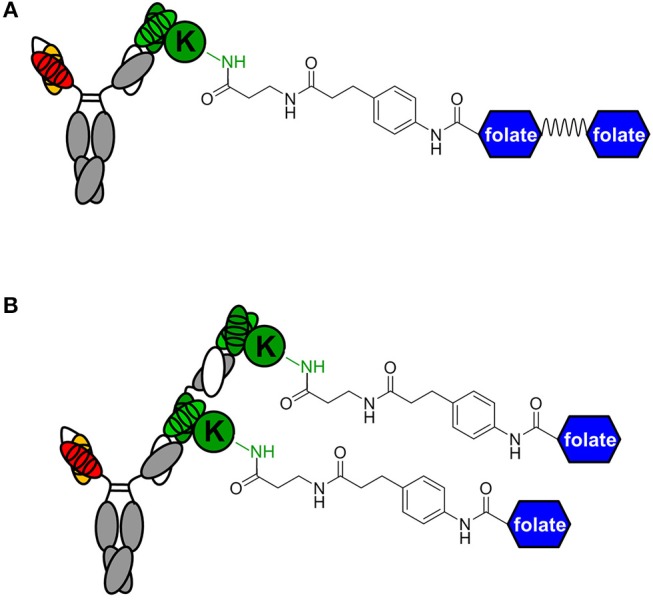
Avidity engineering. In chemically programmed biAbs, the avidity effect of two Fab arms of conventional mAbs can be mimicked by either doubling the chemical or biological component. **(A)** Bivalent FOLR1 targeting with v9 × h38C2_(folate)_2_. The two folate groups are spaced by a flexible PEG linker. **(B)** Bivalent FOLR1 targeting with v9 × (h38C2_folate)_2_. The structural formula shows the amide bond formed by the reaction of β-lactam hapten derivatives of (folate)_2_ and folate, respectively, with the ε-amino group of the uniquely reactive lysine residue (K) in the hapten binding site of h38C2.

While conventional v9 × Farl biAb bound both CD3+ FOLR1– human T-cell line Jurkat and FOLR1+ CD3– human ovarian cancer cell line IGROV-1 as determined by flow cytometry (Figure S2A), v9 × h38C2 biAb only bound Jurkat cells (Figure 2A). To chemically program its h38C2 Fab arm for FOLR1 binding, we first synthesized the trifunctional compounds β-lactam-biotin-folate **1a** and β-lactam-biotin-(folate)_2_ **2** (Figure S3). The β-lactam group mediates highly efficient and selective hapten-driven covalent conjugation to the reactive lysine residue in the hapten binding site of h38C2 (24). The biotin group enables detection. The monovalent and bivalent folate groups in **1a** and **2**, respectively, facilitate FOLR1 binding, in the latter case hypothesized to mimic the avidity effect of two Fab arms of conventional mAbs (Figure 3A). Next, v9 × h38C2 biAb was chemically programmed by incubation with two equivalents (eq) of compound **1a** or **2** in PBS at room temperature (RT) for 4 h. A catalytic assay based on the retro-aldol degradation of methodol by h38C2 (12, 20) revealed nearly complete covalent conjugation of compounds **1a** and **2** at the reactive lysine residue (Figure 2B). Analysis by flow cytometry showed that chemically programmed v9 × h38C2_**1a** and v9 × h38C2_**2** gained the ability to bind IGROV-1 cells while retaining Jurkat cell binding (Figure 2A). Unexpectedly, v9 × h38C2_**1a** and v9 × h38C2_**2** did not show a significant difference with respect to half maximal binding (EC_50_), apparent affinity (K_d_), and maximum number of binding sites (B_max_) (Figure 2C and Table 1). This finding suggested that only one of the two folate groups of compound **2** engaged with FOLR1, precluding the anticipated avidity effect. Notably, these binding parameters were very similar to the EC_50_, K_d_, and B_max_ values determined for the conventional v9 × Farl biAb (Figure S2B and Table 1), suggesting that the biological and chemical monovalent engagement of cell surface FOLR1 by conventional and chemically programmed biAbs, respectively, is comparable.

**Table 1:** Half maximal binding (EC_50_), apparent affinity (K_d_), and maximum number of binding sites (B_max_) of CD3 × FOLR1 biAbs determined by flow cytometry.

**biAb**	**v9 × Farl**	**v9 × h38C2_1a**	**v9 × h38C2_2**	**v9 × h38C2_1b**	**v9 × (h38C2_1b)_**2**_**
EC_50_	17.6 nM	16.7 nM	11.0 nM	34.3 nM	7.4 nM
K_d_	21.7 nM	19.4 nM	12.3 nM	58.0 nM	8.9 nM
B_max_	2,700	3,220	2,990	2,970	1,680

**Figure 4:**
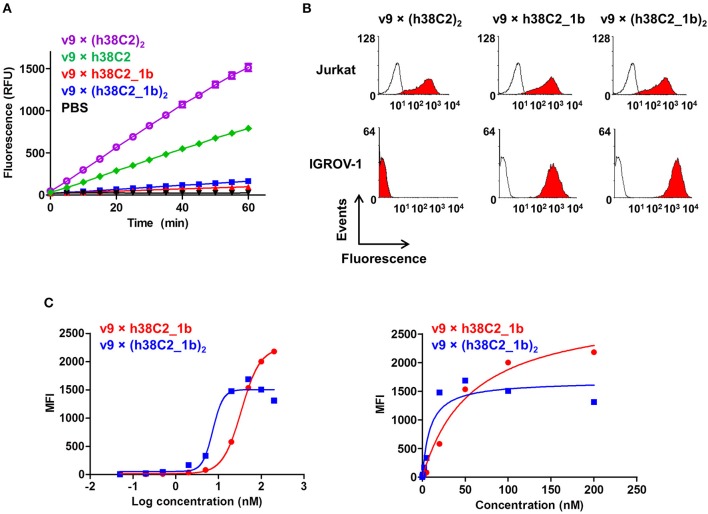
Cell surface binding and catalytic activity of chemically programmed FOLR1-targeting biAb with a tandem Fab arm. **(A)** Catalytic retro-aldol activity of unprogrammed and chemically programmed h38C2-containing biAbs. The signal is reported in relative fluorescent units (RFU; mean ± SD of triplicates). PBS was used as negative control. **(B)** Flow cytometry analysis of unprogrammed and chemically programmed biAbs binding to CD3+ FOLR1– human T-cell line Jurkat and FOLR1+ CD3– human ovarian cancer cell line IGROV-1 using 20 nM biAbs followed by Alexa Fluor 647-conjugated goat anti-human IgG-Fc pAbs. Compound **1b** is a monovalent folate derivative. **(C)** Titration curve of chemically programmed biAbs binding to IGROV-1 cells detected with Alexa Fluor 647-conjugated goat anti-human IgG pAbs (left panel). Saturation analysis of chemically programmed biAbs binding to IGROV-1 cells (right panel). Shown are mean ± SD values from independent triplicates.

The failure of compound **2** to engage FOLR1 bivalently prompted the design of the chemically programmable biAb with a tandem Fab arm (Figure 1C). The (h38C2)_2_ module enables dual chemical programming with compound **1a**, affording an alternative route to avidity engineering (Figure 3B). To test this, we used compound **1b**, which has the same β-lactam, biotin, and folate groups as compound **1a** but differs slightly in their linkage due to a different synthetic route (Figure S3). Chemical programming of v9 × h38C2 to afford v9 × h38C2_**1b**, and v9 × (h38C2)_2_ to afford v9 × (h38C2_**1b**)_2_ was carried out as before. Prior to chemical programming, v9 × (h38C2)_2_ revealed ~2-fold higher catalytic activity than v9 × h38C2, indicating the functional formation of both hapten binding sites in the tandem Fab arm (Figure 4A). After chemical programming, the loss of catalytic activity revealed efficient covalent conjugation of compound **1b** to both biAbs (Figure 4A). Analysis by flow cytometry showed that chemically programmed v9 × h38C2_**1b** and v9 × (h38C2_**1b**)_2_ gained the ability to bind IGROV-1 cells while retaining Jurkat cell binding (Figure 4B). Notably, v9 × (h38C2_**1b**)_2_ revealed >5-fold tighter binding to IGROV-1 cells compared to v9 × h38C2_**1b** with respect to both EC_50_ (7.4 vs. 34 nM) and K_d_ (8.9 vs. 58 nM) (Figure 4C and Table 1). Along with the ~2-fold lower B_max_ of v9 × (h38C2_**1b**)_2_ compared to v9 × h38C2_**1b** (1677 vs. 2966), these binding data collectively suggested that both folate groups of v9 × (h38C2_**1b**)_2_ engaged with FOLR1, revealing successful avidity engineering.

### *In vitro* Activity of FOLR1-Targeting biAbs

**Figure 5:**
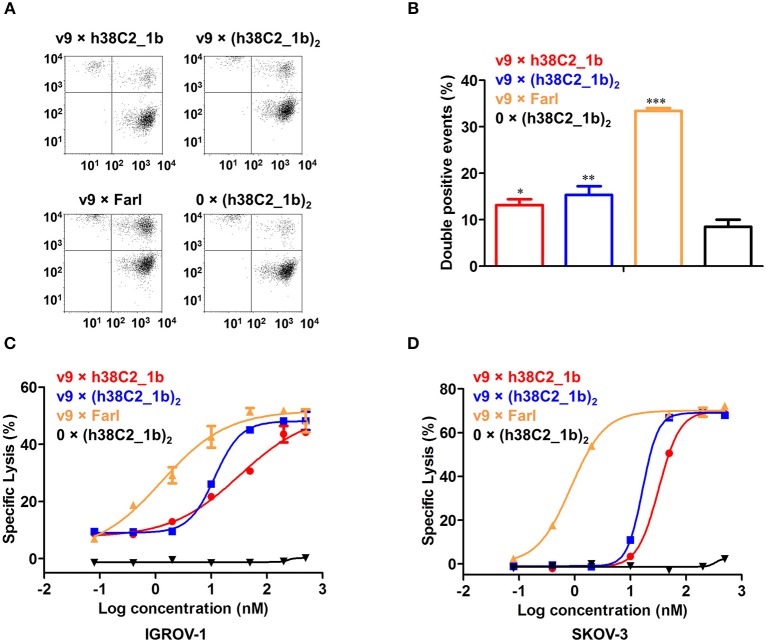
Cross-linking and cytotoxicity mediated by FOLR1-targeting biAbs *in vitro*. **(A)** Cross-linking of 1 × 10^6^ Jurkat cells (stained with CellTrace Far Red) and 2 × 10^5^ IGROV-1 cells (stained with CellTrace CFSE) in the presence of 200 nM FOLR1-targeting biAbs and negative control. Double-stained events were detected by flow cytometry. **(B)** Quantification of double-stained events from three independent triplicates (mean ± SD). An unpaired two-tailed *t*-test was used to analyze significant differences (^*^p < 0.05; ^**^p < 0.01; ^***^p < 0.001). **(C)** Cytotoxicity of biAbs tested with *ex vivo* expanded primary human T cells (effector cells) and IGROV-1 cells (target cells) at an effector-to-target cell ratio of 10:1 and measured after 16-h incubation. Shown are mean ± SD values from independent triplicates. Chemically programmed biAb v9 × h38C2_**1b** (red circles) was significantly (*p* < 0.001; extra sum-of-squares F-test) less potent than chemically programmed biAb v9 × (h38C2_**1b**)_2_ (blue squares) and conventional biAb v9 × Farl (yellow triangles). **(D)** Corresponding cytotoxicity toward SKOV-3 cells. Shown are mean ± SD values from independent triplicates. Again, chemically programmed biAb v9 × h38C2_**1b** (red circles) was significantly (*p* < 0.001; extra sum-of-squares F-test) less potent than chemically programmed biAb v9 × (h38C2_**1b**)_2_ (blue squares) and conventional biAb v9 × Farl (yellow triangles). The negative control, 0 × (h38C2_**1b**)_2_, revealed no cytotoxicity in either experiment.

To examine whether the three FOLR1-targeting biAbs can recruit effector cells to target cells, we first analyzed their cross-linking capability by flow cytometry. Jurkat cells stained red served as effector cells and IGROV-1 cells stained green as target cells. The two cell populations were mixed at an effector-to-target cell ratio of 5:1 in the presence of 200 nM biAbs for 2 h. Double-stained events detected and quantified by flow cytometry, indicated cross-linked effector and target cells. Both conventional and chemically programmed biAbs showed significantly higher cross-linking between Jurkat cells and IGROV-1 cells compared with a negative control, 0 × (h38C2_**1b**)_2_, without the CD3-binding arm (Figures 5A,B). While the conventional biAb, v9 × Farl, mediated significantly more cross-linking than both chemically programmed biAbs, v9 × h38C2_**1b** and v9 × (h38C2_**1b**)_2_, the higher avidity of the latter did not translate to greater cross-linking capability in this experiment (Figure 5B).

Next, we compared the three FOLR1-targeting biAbs with respect to eliciting effector cell-mediated target cell killing. Primary human T cells that had been expanded from healthy donor peripheral blood mononuclear cells (PBMCs) *ex vivo*, served as effector cells and FOLR1-expressing IGROV-1 and SKOV-3 cells as target cells. Analysis by flow cytometry showed that the expanded primary human T cells consisted of 67% CD4+ and 32% CD8+ T cells (Figure S4). Specific lysis of target cells after 16-h incubation at an effector-to-target cell ratio of 10:1 was assessed in the presence of a range of concentrations of biAbs. As shown in Figure 5C, both chemically programmed biAbs, v9 × h38C2_**1b** and v9 × (h38C2_**1b**)_2_ revealed selective and potent killing of IGROV-1 cells with EC_50_ values of 33 and 12 nM, respectively. The conventional biAb, v9 × Farl, was significantly more potent with an EC_50_ of 1.3 nM. Similar results were observed when SKOV-3 cells were used as target cells, with EC_50_ values of 32 nM, 17 nM, and 0.86 nM for v9 × h38C2_**1b**, v9 × (h38C2_**1b**)_2_, and v9 × Farl, respectively (Figure 5D). Negative control 0 × (h38C2_**1b**)_2_ was inactive in both experiments (Figures 5C,D).

**Figure 6:**
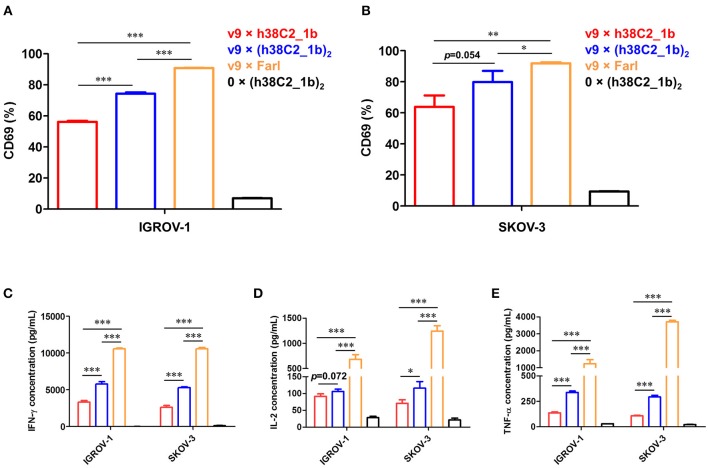
T-cell activation mediated by FOLR1-targeting biAbs *in vitro*. *Ex vivo* expanded primary human T cells (effector cells) were incubated with 50 nM of the indicated biAbs in the presence of IGROV-1 or SKOV-3 cells (target cells) at an effector-to-target cell ratio of 10:1 for 16 h. Shown in the upper panel is the percentage of activated T cells based on CD69 expression after incubation with IGROV-1 **(A)** or SKOV-3 cells **(B)** measured by flow cytometry. Shown in the lower panel is the cytokine release measured by ELISA for IFN-γ **(C)**, IL-2 **(D)** and TNF-α **(E)**. All experiments are shown as mean ± SD values from independent triplicates. An unpaired two-tailed *t*-test was used to analyze significant differences (^*^p < 0.05; ^**^p < 0.01; ^***^p < 0.001).

Using a defined concentration of 50 nM, the ability of the biAbs to mediate primary T-cell activation after 16-h incubation with IGROV-1 and SKOV-3 cells was analyzed. Robust upregulation of early T-cell activation marker CD69 was detected by flow cytometry for all three FOLR1-targeting biAbs and both ovarian cancer cell lines when compared to the negative control (Figures 6A,B). Equally robust upregulation of the secretion of type I cytokines IFN-γ, IL-2, and TNF-α was observed by ELISA (Figures 6C–E). In agreement with the cytotoxicity data, the conventional biAb revealed significantly greater T-cell activation than the chemically programmed biAbs. Among these, avidity engineered v9 × (h38C2_**1b**)_2_ was consistently more active than v9 × h38C2_**1b**.

### *In vivo* Activity of FOLR1-Targeting biAbs

**Figure 7:**
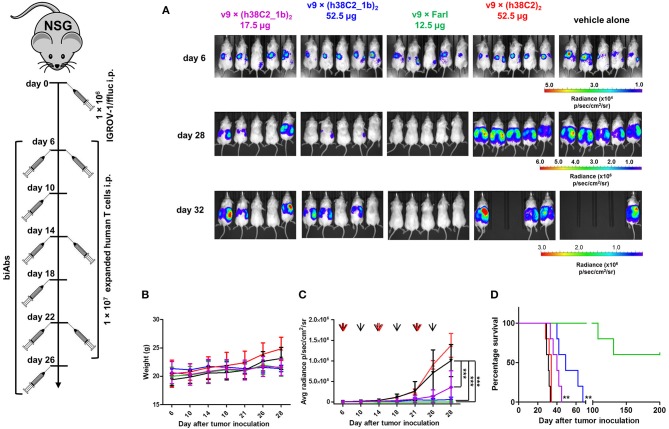
*In vivo* activity of FOLR1-targeting biAbs. Five cohorts of mice (*n* = 5) were inoculated with 1 × 10^6^ IGROV-1/ffluc cells via i.p. injection. After 6 days, 1 × 10^7^ *ex vivo* expanded primary human T cells and the indicated biAbs or vehicle alone (PBS) were administered by the same route. The mice received a total of three doses of T cells every 8 days and a total of six doses of biAbs or vehicle alone every 4 days. **(A)** Bioluminescence images of all 25 mice from day 6 (before treatment) and days 28 and 32 (after treatment) are shown. **(B)** The weight of all 25 mice was recorded on the indicated days (mean ± SD). **(C)** Starting on day 6, all 25 mice were imaged every 3–5 days and their radiance was recorded (mean ± SD). Significant differences between cohorts treated with biAbs (colored graphs) and vehicle alone (black graph) were calculated using an unpaired two-tailed *t*-test (^***^p < 0.001). Red arrows indicate the three T-cell doses and black arrows the six biAb or vehicle alone doses. **(D)** Corresponding Kaplan-Meier survival curves with *p*-values using log-rank (Mantel-Cox) test (^**^p < 0.01).

The robust *in vitro* activity of our FOLR1-targeting biAbs prompted us to next study their performance *in vivo*. For this, we used a mouse model of human ovarian cancer that is based on injecting IGROV-1 cells transfected with firefly luciferase (ffluc) intraperotoneally (i.p.) into NOD/SCID/IL-2Rγ^null^ (NSG) mice (10). Intraperitoneal xenografts of IGROV-1 cells in immunodeficient mice mimic the human disease with respect to carcinomatosis and ascites and have been widely used for preclinically assessing ovarian cancer therapeutics. Five cohorts of five female NSG mice each were injected i.p. with 1 × 10^6^ IGROV-1/ffluc cells. After six days, the mice were injected i.p. with 1 × 10^7^ *ex vivo* expanded primary human T cells. One hour later, 17.5 μg (cohort 1) and 52.5 μg (cohort 2) of v9 × (h38C2_**1b**)_2_, 12.5 μg of the v9 × Farl (cohort 3; same molar dosage as used for cohort 1), 52.5 μg of unprogrammed v9 × (h38C2)_2_ (control cohort 4), and vehicle (PBS) alone (control cohort 5) were administered i.p. in a 100 μL volume. All 5 cohorts were treated with a total of three doses of T cells (every 8 days) and six doses of biAbs or PBS (every 4 days). Mice were pre-conditioned with 250 μL human serum 24 h before every dose (25). Note that all experiments were conducted without reducing endogenous folate concentrations in the mice. To assess the response to the treatment, *in vivo* bioluminescence imaging was performed prior to the first dose and then every 3–5 days until day 32 when the mice in the control cohorts 4 and 5 started to die due to aggressive tumor growth (Figure 7). In cohort 3, which received the conventional biAb, we observed complete tumor eradication after one dose of T cells and two doses of biAbs (Figures 7A,C). Treatment with the higher dosage of the chemically programmed biAb (cohort 2) robustly decreased tumor burden in the 1st week and significantly stalled further tumor growth (Figures 7A,C). The lower dosage (cohort 1) revealed significant tumor growth retardation but relapsed immediately when the treatment ceased (Figures 7A,C). No weight loss or other obvious signs of toxicity were observed during the treatment with the conventional or chemically programmed biAbs (Figure 7B). Kaplan–Meier survival curves revealed that both lower (*p* < 0.001) and higher (*p* < 0.001) dosage of the chemically programmed biAb, but not the unprogrammed biAb, significantly prolonged survival when compared to vehicle alone (Figure 7D). Notably, mice treated with the conventional biAb survived much longer than all other cohorts and 60% of the mice were still alive with no evidence of relapse when the experiment was terminated on day 200.

We next carried out a PK study with v9 × h38C2_**1b**, v9 × (h38C2_**1b**)_2_, and v9 × Farl in mice to examine their circulatory half-lives. For each biAb, four female CD-1 mice were injected i.p. with 6 mg/kg. Blood was withdrawn from the tail vein at nine time points (5 min to 336 h after injection) and plasma was prepared. The functional concentrations of chemically programmed or conventional biAbs in the plasma was measured by analyzing the binding to IGROV-1 cells by flow cytometry. Analysis of the PK parameters by two-compartment modeling revealed t_1/2_ values (mean ± SD) of v9 × h38C2_**1b**, v9 × (h38C2_**1b**)_2_, and v9 × Farl, respectively, of 31.1 ± 9.9, 32.2 ± 14.3 h, and 200.6 ± 82.2 h (Figures S5A–C and Table 2). To ensure that the shorter circulatory half-lives of the chemically programmed biAbs were not due to instability of the conjugates, we incubated v9 × h38C2_**1b**, v9 × (h38C2_**1b**)_2_, and v9 × Farl for 4 days in mouse plasma at 37°C. Subsequent flow cytometry analysis with IGROV-1 cells revealed no loss of binding following the incubation (Figure S5D), confirming the high stability of all three FOLR1-targeting biAbs.

**Table 2:** Pharmacokinetic parameters of v9 × h38C2_**1b** (top row), v9 × (h38C2_**1b**)_2_ (middle row), and v9 × Farl (bottom row)^a^.

**t½ (h)**	**AUC (mg/mL × h)**	**CL (mL/h/kg)**
31.1 ± 9.9	2.8 ± 1.2	2.7 ± 1.7
32.2 ± 14.3	2.4 ± 1.2	3.5 ± 2.8
200.6 ± 82.2	21.9 ± 1.2	0.18 ± 0.03

a *Mean ± SD values from 4 mice*.

### *In vitro* Activity of Chemically Programmed biAbs That Target Integrin α_4_β_1_

For independent proof-of-concept that the chemical programming of v9 × h38C2 and v9 × (h38C2)_2_ can mediate the redirection of cytotoxic T cells, we used a different compound (LLP2A) to a different molecular (integrin α_4_β_1_) and cellular (JeKo-1, a mantle cell lymphoma cell line) target. LLP2A is a peptidomimetic that was selected previously from a one-bead-one-compound chemical library for binding to integrin α_4_β_1_ (26). Notably, the LLP2A binding site is only exposed when integrin α_4_β_1_ is in its open conformation which is found on malignant B cells and in other cancers. To enable chemical programming with LLP2A, we synthesized β-lactam-biotin-LLP2A **3** (Figure S3). Chemical programming of v9 × h38C2 and v9 × (h38C2)_2_ with compound **3** was carried out as before. Loss of catalytic activity revealed efficient covalent conjugation of compound **3** to both biAbs (Figure S6A). As determined by ELISA, chemically programmed biAbs v9 × h38C2_**3** and v9 × (h38C2_**3**)_2_, but not the unconjugated biAb, selectively bound to immobilized integrin α_4_β_1_ in the presence of 1 mM Mn^2+^, which stabilizes the open conformation (Figure S6B). Moreover, a comparison of chemically programmed biAbs v9 × h38C2_**3b** and v9 × (h38C2_**3b**)_2_ clearly revealed successful avidity engineering through the tandem Fab arm (Figure S6B). In addition, as shown in Figure S6C, both v9 × h38C2_**3** and v9 × (h38C2_**3**)_2_ revealed selective and potent killing of JeKo-1 cells which constitutively express integrin α_4_β_1_ in its open conformation. Again, the avidity engineered v9 × (h38C2_**3**)_2_ was significantly more potent than v9 × h38C2_**3** with EC_50_ values of 7.8 and 23.6 nM, respectively. Negative control 0 × (h38C2_**3**)_2_ was inactive (Figure S6C).

## Discussion

Building on the properties and modularity of the natural antibody molecule, engineered antibody molecules with properties and structures not found in nature have emerged among FDA-approved therapeutic mAbs, including biAbs blinatumomab (Blincyto^®^) (3) for cancer therapy and emicizumab*-*kxwh (Hemlibra^®^) (27) for hemophilia treatment. While all biAbs are defined by their ability to bind two antigens, blinatumomab is a biAb that cross-links effector and target cells for cancer immunotherapy by engaging CD3 on T cells and CD19 on acute lymphoblastic leukemia cells. Many such biAbs are currently at various stages of preclinical and clinical development. By activating and recruiting T-cells *in situ*, cancer immunotherapeutic biAbs do not require the logistically challenging steps of GMP manufacturing of chimeric antigen receptor T-cell therapy (CAR-T) (28) and have remained highly competitive (1, 29, 30).

Although collectively three biAb and CAR-T therapeutics have been approved by the FDA for the treatment of leukemia and lymphoma, their therapeutic utility for solid malignancies, which make up 90% of all cancers, remains to be established clinically. A major impediment is the immunosuppressive tumor environment that counteracts T-cell infiltration, activation, and recruitment. Another challenge is the identification of cell surface receptors that are selectively expressed on tumor cells and can serve as targets for biAbs and CAR-Ts that do not harm healthy cells and tissues. Although FOLR1, like the vast majority of cell surface receptors, is not exclusively expressed on tumor cells, its expression profile in ovarian and lung cancer patients is considered conductive for FOLR1-targeting interventions such as mAbs, antibody-drug conjugates (ADCs), and small molecule drug conjugates, all of which were generally tolerated in clinical trials (31–33). The most promising among these is the ADC mirvetuximab soravtansine, which is in a randomized multicenter phase III clinical trial for women with chemotherapy-resistant FOLR1+ ovarian cancer (34, 35). By contrast, mAb farletuzumab in combination with chemotherapy did not reveal an improvement in progression free survival compared to chemotherapy alone (31).

Based on these clinical trial data and the advances made with biAb engineering (36), FOLR1-targeting T-cell engaging biAbs, which were among the first clinically translated biAbs generated by hybrid hybridoma technology and limited by human anti-mouse antibodies in cancer patients (37), warrant renewed preclinical and clinical investigations as potent and safe alternative treatment modalities, possibly in combination with immune checkpoint inhibitors to overcome T-cell dysfunction in solid malignancies (38). Given the fact that FOLR1 can be targeted with both mAbs and small molecules, i.e., folate and folate derivatives, it also provides an opportunity to compare conventional biAbs to the concept of chemically programmed biAbs that combine an invariable biological component with a variable chemical component. An adaptation of chemically programmed antibodies (11), chemically programmed biAbs endow natural or synthetic small molecules that target cell surface receptors with the power of cancer immunotherapy (7, 10). The natural small molecule folate, which has been extensively utilized for the selective delivery of diagnostic and therapeutic cargos to FOLR1+ cancer cells *in vivo* (39), represents an ideal chemical component to test the concept of chemically programmed biAbs side-by-side with a conventional biAb that uses an anti-human FOLR1 mAb.

While previous studies with chemically programmed CD3 × FOLR1 biAbs employed antibody fragments with short circulatory half-lives requiring daily dosing (9, 10), one objective of the current study was to generate and evaluate biAb formats that contain an Fc module of human IgG1 for prolonged circulatory half-lives and less frequent dosing. To do so, we built three asymmetric biAb formats that afford different strategies for monovalent and bivalent FOLR1 binding, while having monovalent CD3 engagement in common. This design along with the removal of the N-glycosylation site in the Fc module, which is required for Fcγ receptor binding, eludes the systemic activation of CD3+ T cells in the absence of FOLR1+ cancer cells. Bivalent FOLR1 binding by the chemically programmed biAb was pursued by chemically programming either a single h38C2 Fab arm with a bivalent folate derivative or a tandem h38C2 Fab arm with a monovalent folate derivative. The latter but not the former revealed successful avidity engineering in binding assays, presumably due to greater spacing, flexibility, and accessibility of the two folate groups. The cell surface density of FOLR1, which is heterogeneous in any given tumor and between different tumors, is likely another critical parameter of the avidity effect that should be taken into consideration. Comparing *in vitro* cross-linking of FOLR1+ cancer cells and CD3+ T cells, cancer cell killing, and T-cell activation, bivalent FOLR1 binding significantly outperformed monovalent FOLR1 binding by chemically programmed biAbs. The dual folate displaying biAb also revealed potent dose-dependent activity in a mouse model of ovarian cancer with significantly prolonged survival compared to the non-displaying biAb. However, the conventional biAb, which revealed similar affinity for FOLR1 as the single folate displaying biAb and less avidity than the dual folate displaying biAb, was the most potent reagent *in vitro* and *in vivo*. Remarkably, it cured 60% of mice with established and aggressive ovarian cancer.

What are possible reasons for the conventional biAb outperforming the chemically programmed biAbs? First, it is important to note that, unlike the conventional biAb, the chemically programmed biAbs compete with folate for FOLR1 binding. Our *in vitro* cytotoxicity assays were carried out in X-VIVO 20 medium supplemented with 5% (v/v) human serum, both of which contain folate. *In vivo*, the chemically programmed biAbs compete with endogenous folate in the blood (~250 nM in laboratory mice and ~20 nM in humans) for FOLR1 binding (40) and also bind to mouse folate receptors. In contrast, the conventional biAb only saw FOLR1 on the xenografted human ovarian cancer cells. Thus, the *in vivo* performance of the chemically programmed biAb in the mouse model may provide a more realistic assessment of the potential of CD3 × FOLR1 biAbs in ovarian cancer patients. Second, our PK study revealed much shorter circulatory half-lives (~30 h) for the two chemically programmed biAbs compared to the conventional biAb (~200 h) following i.p. injection. *In vitro* experiments showed that this difference was not due to instability of the conjugate in mouse plasma. Notably, folate-based small molecule drug conjugates are known to be rapidly removed from circulation (41). Again, due to their interaction with the host system, the chemically programmed biAbs may provide a more realistic assessment of the PK parameters of CD3 × FOLR1 biAbs. Third, additional factors inherent to the interaction of farletuzumab and folate with FOLR1 may be at play. Despite their comparable affinities, similar internalization rates (42, 43), and neighboring epitopes (44), the stability and kinetics of cytolytic synapse formation (45) warrant further investigation.

While folate is a suitable natural small molecule for demonstrating proof-of-concept of chemically programmed biAbs targeting FOLR1 in ovarian cancer, the crystal structures of FOLR1 and FOLR1 in complex with folate revealed extensive opportunities for developing synthetic small molecule derivatives of folate that, by binding FOLR1 with higher specificity and affinity, can compete better with endogenous folate (46, 47). As such, these derivatives are of exceptional interest to fine-tuning chemically programmed biAbs. Conventional biAbs can also be fine-tuned by varying the FOLR1-binding Fab arm, but this requires the cloning, expression, and purification of a new protein for each variant. Chemically programmed biAbs, by contrast, only require one protein to test a virtually unlimited number of variants (12). To demonstrate this versatility, we included a synthetic small molecule for chemical programming in the current study. LLP2A is a peptidomimetic derived by chemical library screening for high affinity and specificity to the open conformation of integrin α_4_β_1_ (26). As such, LLP2A cannot directly be compared to a mAb and thus signifies opportunities for chemically programmed biAbs that have no conventional biAb counterpart and competitor.

Substantial effort has been devoted to the screening of actual and virtual small molecule libraries to identify targetable and druggable binding sites of proteins including cell surface receptors, collectively known as the pocketome (48). Venturing deep into chemical space, these synthetic small molecules have the potential to challenge natural recognition repertoires including antibody molecules in terms of specificity and affinity. We consider our work on chemically programmed biAbs a complementing effort that can endow synthetic small molecules targeting the cell surface pocketome with prolonged circulatory half-life, higher avidity, and the ability to recruit the immune system.

## Data Availability

All datasets generated for this study are included in the manuscript and/or the Supplementary Files.

## Ethics Statement

This study was carried out in accordance with the recommendations of the NIH Guide for the Care and Use of Laboratory Animals. The protocol was approved by the Institutional Animal Care and Use Committee of The Scripps Research Institute.

## Author Contributions

JQ and CR conceived the study and wrote the manuscript. JQ performed the experiments and analyzed the data under supervision and guidance of CR. DH and CN synthesized the folate derivatives under supervision and guidance of TB. All authors reviewed the manuscript.

### Conflict of Interest Statement

The authors declare that the research was conducted in the absence of any commercial or financial relationships that could be construed as a potential conflict of interest.

## References

[1] Ellerman D. Bispecific T-cell engagers: towards understanding variables influencing the *in vitro* potency and tumor selectivity and their modulation to enhance their efficacy and safety. Methods. (2019) 154:102–17. 10.1016/j.ymeth.2018.10.026 30395966

[2] Clynes RA Desjarlais JR. Redirected T cell cytotoxicity in cancer therapy. Annu Rev Med. (2019) 70:437–50 10.1146/annurev-med-062617-035821 30379598

[3] Przepiorka D Ko CW Deisseroth A Yancey CL Candau-Chacon R Chiu HJ. FDA approval: blinatumomab. Clin Cancer Res. (2015) 21:4035–9. 10.1158/1078-0432.CCR-15-0612 26374073

[4] Jen EY Xu Q Schetter A Przepiorka D Shen YL Roscoe D. FDA approval: blinatumomab for patients with B-cell precursor acute lymphoblastic leukemia in morphologic remission with minimal residual disease. Clin Cancer Res. Cancer Res. (2019) 25:473–7. 10.1158/1078-0432.CCR-18-2337 30254079

[5] Kantarjian H Stein A Gokbuget N Fielding AK Schuh AC Ribera JM. Blinatumomab versus chemotherapy for advanced acute lymphoblastic leukemia. N Engl J Med. (2017) 376:836–47. 10.1056/NEJMoa1609783 28249141 PMC5881572

[6] Wu X Demarest SJ. Building blocks for bispecific and trispecific antibodies. Methods. (2019) 154:3–9. 10.1016/j.ymeth.2018.08.010 30172007

[7] Cui H Thomas JD Burke TR Jr Rader C. Chemically programmed bispecific antibodies that recruit and activate T cells. J Biol Chem. (2012) 287:28206–14. 10.1074/jbc.M112.384594 22761439 PMC3436515

[8] Kim CH Axup JY Lawson BR Yun H Tardif V Choi SH. Bispecific small molecule-antibody conjugate targeting prostate cancer. Proc Natl Acad Sci Natl Acad Sci USA. (2013) 110:17796–801. 10.1073/pnas.1316026110 24127589 PMC3816437

[9] Kularatne SA Deshmukh V Gymnopoulos M Biroc SL Xia J Srinagesh S. Recruiting cytotoxic T cells to folate-receptor-positive cancer cells. Angew Chem Int Ed Engl. (2013) 52:12101–4. 10.1002/anie.201306866 24573789 PMC4012751

[10] Walseng E Nelson CG Qi J Nanna AR Roush WR Goswami RK. Chemically programmed bispecific antibodies in diabody format. J Biol Chem. (2016) Chem. (2016) 291:19661–73. 10.1074/jbc.M116.745588 27445334 PMC5016699

[11] Rader C. Chemically programmed antibodies. Trends Biotechnol. (2014);32:186–97. 10.1016/j.tibtech.2014.02.003 24630478 PMC3978777

[12] Rader C Turner JM Heine A Shabat D Sinha SC Wilson IA. A humanized aldolase antibody for selective chemotherapy and adaptor immunotherapy. J Mol Biol. (2003) 332:889–99. 10.1016/S0022-2836(03)009926 12972259

[13] Wagner J Lerner RA Barbas CF III. Efficient aldolase catalytic antibodies that use the enamine mechanism of natural enzymes. Science. (1995) 270:1797–800. 8525368 10.1126/science.270.5243.1797

[14] Barbas CF III Heine A Zhong G Hoffmann T Gramatikova S Bjornestedt R. Immune versus natural selection: antibody aldolases with enzymic rates but broader scope. Science. (1997) 278:2085–92. 9405338 10.1126/science.278.5346.2085

[15] Zhu X Tanaka F Lerner RA Barbas CF III Wilson IA. Direct observation of an enamine intermediate in amine catalysis. J Am Chem Soc. (2009) 131:18206–7. 10.1021/ja907271a 19968282 PMC3227542

[16] Rader C Sinha SC Popkov M Lerner RA Barbas CF III. Chemically programmed monoclonal antibodies for cancer therapy: adaptor immunotherapy based on a covalent antibody catalyst. Proc Natl Acad Sci USA. (2003) (2003) 100:5396–400. 10.1073/pnas.0931308100 12702756 PMC154356

[17] Antony AC. Folate receptors. Annu Rev Nutr. (1996) 16:501–21. 10.1146/annurev.nu.16.070196.002441 8839936

[18] Xia W Low PS. Folate-targeted therapies for cancer. J Med Chem. (2010) 53:6811–24. 10.1021/jm100509v 20666486

[19] Fernandez M Javaid F Chudasama V. Advances in targeting the folate receptor in the treatment/imaging of cancers. Chem Sci. (2018) 9:790–810. 9:790–810. 10.1039/c7sc04004k 29675145 PMC5890329

[20] List B Barbas CF III Lerner RA. Aldol sensors for the rapid generation of tunable fluorescence by antibody catalysis. Proc Natl Acad Sci USA. (1998) 95:15351–5. 9860972 10.1073/pnas.95.26.15351 PMC28046

[21] Qi J Li X Peng H Cook EM Dadashian EL Wiestner A. Potent and selective antitumor activity of a T cell-engaging bispecific antibody targeting a membrane-proximal epitope of ROR1. Proc Natl Acad Sci USA. (2018) USA. (2018) 115:E5467–76. 10.1073/pnas.1719905115 29844189 PMC6004433

[22] Merchant AM Zhu Z Yuan JQ Goddard A Adams CW Presta LG. An efficient route to human bispecific IgG. Nat Biotechnol. (1998) 16:677–81. 10.1038/nbt0798-677 9661204

[23] Nanna AR Li X Walseng E Pedzisa L Goydel RS Hymel D. Harnessing a catalytic lysine residue for the one-step preparation of homogeneous antibody-drug conjugates. Nat Commun. (2017) 8:1112. 10.1038/s41467-017-01257-1 29062027 PMC5653646

[24] Gavrilyuk JI Wuellner U Barbas CF III. Beta-lactam-based approach for the chemical programming of aldolase antibody 38C2. Bioorg Med Chem Lett. Lett. (2009) 19:1421–4. 10.1016/j.bmcl.2009.01.028 19181522 PMC2688461

[25] Li F Ulrich ML Shih VF Cochran JH Hunter JH Westendorf L. Mouse strains influence clearance and efficacy of antibody and antibody-drug conjugate via Fc-FcgammaR interaction. Mol Cancer Ther. (2019)18:780–7 (2019)18:780–7 10.1158/1535-7163.MCT-18-0977 30824607

[26] Peng L Liu R Marik J Wang X Takada Y Lam KS. Combinatorial chemistry identifies high-affinity peptidomimetics against alpha4beta1 integrin for *in vivo* tumor imaging. Nat Chem Biol. (2006) 2:381–9. 10.1038/nchembio798 16767086

[27] Ragni MV. Mimicking Factor VIII to manage the Factor VIII-deficient state. N Engl J Med. (2018) 379:880–2. 10.1056/NEJMe1808789 30157395

[28] Kohl U Arsenieva S Holzinger A Abken H. CAR T cells in trials: recent achievements and challenges that remain in the production of modified T cells for clinical applications. Hum Gene Ther. (2018) 29:559–68. 10.1089/hum.2017.254 29620951

[29] Aldoss I Bargou RC Nagorsen D Friberg GR Baeuerle PA Forman SJ. Redirecting T cells to eradicate B-cell acute lymphoblastic leukemia: bispecific T-cell engagers and chimeric antigen receptors. Leukemia. (2017) 31:777–87. 10.1038/leu.2016.391 28028314

[30] Slaney CY Wang P Darcy PK Kershaw MH. CARs versus BiTEs: a comparison between T cell-redirection strategies for cancer treatment. Cancer Discov. (2018) 8:924–34. 10.1158/2159-8290.CD-18-0297 30012854

[31] Vergote I Armstrong D Scambia G Teneriello M Sehouli J Schweizer C. A randomized, double-blind, placebo-controlled, Phase III study to assess efficacy and safety of weekly farletuzumab in combination with carboplatin and taxane in patients with ovarian cancer in first platinum-sensitive relapse. J Clin Oncol. (2016) 34:2271–8. 10.1200/JCO.2015.63.2596 27001568

[32] Moore KN Martin LP O'Malley DM Matulonis UA Konner JA Perez RP. Safety and activity of mirvetuximab soravtansine (IMGN853), a folate receptor alpha-targeting antibody-drug conjugate, in platinum-resistant ovarian, fallopian tube, or primary peritoneal cancer: a Phase I expansion study. J Clin J Clin Oncol. (2017) 35:1112–8. 10.1200/JCO.2016.69.9538 28029313 PMC5559878

[33] Lorusso PM Edelman MJ Bever SL Forman KM Pilat M Quinn MF. Phase I study of folate conjugate EC145 (Vintafolide) in patients with refractory solid tumors. J Clin Oncol. (2012) 30:4011–6. 10.1200/JCO.2011.41.4946 23032618 PMC4104289

[34] Moore KN Vergote I Oaknin A Colombo N Banerjee S Oza A. FORWARD I: a Phase III study of mirvetuximab soravtansine versus chemotherapy in platinum-resistant ovarian cancer. Future Oncol. (2018) 14:1669–78. 10.2217/fon-2017-0646 29424243

[35] Kaplon H Reichert JM. Antibodies to watch in 2019. mAbs. (2019) 11:219–38. 10.1080/19420862.2018.1556465 30516432 PMC6380461

[36] Rader C “One, if by land, and two, if by sea”: bispecific antibodies join the revolution. Methods. (2019) 154:1–2. 10.1016/j.ymeth.2018.12.004 30683271

[37] Bolhuis RL Lamers CH Goey SH Eggermont AM Trimbos JB Stoter G. Adoptive immunotherapy of ovarian carcinoma with bs-MAb-targeted lymphocytes: a multicenter study. Int J Cancer Suppl. (1992) 7:78–81 1428412

[38] Schreiner J Thommen DS Herzig P Bacac M Klein C Roller A. Expression of inhibitory receptors on intratumoral T cells modulates the activity of a T cell-bispecific antibody targeting folate receptor. Oncoimmunology. (2016) 5:e1062969. 10.1080/2162402X.2015.1062969 27057429 PMC4801463

[39] Low PS Kularatne SA. Folate-targeted therapeutic and imaging agents for cancer. Curr Opin Chem Biol. (2009) 13:256–62. 10.1016/j.cbpa.2009.03.022 19419901

[40] Leamon CP Reddy JA Dorton R Bloomfield A Emsweller K Parker N. Impact of high and low folate diets on tissue folate receptor levels and antitumor responses toward folate-drug conjugates. J Pharmacol Exp Ther. Ther. (2008) 327:918–25. 10.1124/jpet.108.143206 18791065

[41] Leamon CP Parker MA Vlahov IR Xu LC Reddy JA Vetzel M. Synthesis and biological evaluation of EC20: a new folate-derived, (99m)Tc-based radiopharmaceutical. Bioconjug Chem. (2002) 13:1200–10. 13:1200–10. 10.1021/bc0200430 12440854

[42] Smith-Jones PM Pandit-Taskar N Cao W O'Donoghue J Philips MD Carrasquillo J. Preclinical radioimmunotargeting of folate receptor alpha using the monoclonal antibody conjugate DOTA-MORAb-003. Nucl Med Biol. (2008) Med Biol. (2008) 35:343–51. 10.1016/j.nucmedbio.2007.12.008 18355690 PMC2676680

[43] O'Shannessy DJ Somers EB Albone E Cheng X Park YC Tomkowicz BE. Characterization of the human folate receptor alpha via novel antibody-based probes. Oncotarget. (2011) 2:1227–43. 10.18632/oncotarget.412 22204844 PMC3282080

[44] Jenkins MR Griffiths GM. The synapse and cytolytic machinery of cytotoxic T cells. Curr Opin Immunol. (2010) 22:308–13. 10.1016/j.coi.2010.02.008 20226643 PMC4101800

[45] Chen C Ke J Zhou XE Yi W Brunzelle JS Li J. Structural basis for molecular recognition of folic acid by folate receptors. Nature. (2013) 500:486–9. 10.1038/nature12327 23851396 PMC5797940

[46] Wibowo AS Singh M Reeder KM Carter JJ Kovach AR Meng W. Structures of human folate receptors reveal biological trafficking states and diversity in folate and antifolate recognition. Proc Natl Acad Sci USA. (2013) USA. (2013) 110:15180–8. 10.1073/pnas.1308827110 23934049 PMC3780903

[47] Paulos CM Reddy JA Leamon CP Turk MJ Low PS. Ligand binding and kinetics of folate receptor recycling *in vivo*: impact on receptor-mediated drug delivery. Mol Pharmacol. (2004) 66:1406–14. 10.1124/mol.104.003723 15371560

[48] Bhagavat R Sankar S Srinivasan N Chandra N. An augmented pocketome: detection and analysis of small-molecule binding pockets in proteins of known 3D structure. Structure. (2018) 26:499–512. 10.1016/j.str.2018.02.001 29514079

