# Supplementary material for: Conventional and Chemically Programmed Asymmetric Bispecific Antibodies Targeting Folate Receptor 1
Source: Front Immunol. 2019 Aug 21;10:1994. doi: 10.3389/fimmu.2019.01994 (PMC6712926; doi:10.3389/fimmu.2019.01994)
Supplement: Supplementary file 1 [file Data_Sheet_1.docx]

**SUPPLEMENTARY INFORMATION**

**Conventional and Chemically Programmed** **Asymmetric Bispecific Antibodies Targeting Folate Receptor 1**

***Junpeng Qi^1^, David Hymel^2^, Christopher G. Nelson^2^, Terrence R. Burke, Jr.^2^, and Christoph Rader^1*^***

*^1^Department of Immunology and Microbiology, The Scripps Research Institute, Jupiter, FL, United States, ^2^Chemical Biology Laboratory, Center for Cancer Research, National Cancer Institute, National Institutes of Health, Frederick, MD, United States*

***^*^Correspondence:***

*Christoph Rader*

[*crader@scripps.edu*](mailto:crader@scripps.edu)

**SUPPLEMENTARY MATERIALS AND METHODS**

**Amino acid sequences**

*v9 V_H_-(G_4_S)_3_-V_L_-hinge-C_H_2-C_H_3 (holes) polypeptide:*

EVQLVESGGGLVQPGGSLRLSCAASGYSFTGYTMNWVRQAPGKGLEWVALINPYKGVSTYNQKFKDRFTISVDKSKNTAYLQMNSLRAEDTAVYYCARSGYYGDSDWYFDVWGQGTLVTVSSGGGGSGGGGSGGGGSDIQMTQSPSSLSASVGDRVTITCRASQDIRNYLNWYQQKPGKAPKLLIYYTSRLESGVPSRFSGSGSGTDYTLTISSLQPEDFATYYCQQGNTLPWTFGQGTKVEIKEPKSSDKTHTCPPCPAPELLGGPSVFLFPPKPKDTLMISRTPEVTCVVVDVSHEDPEVKFNWYVDGVEVHNAKTKPREEQYASTYRVVSVLTVLHQDWLNGKEYKCKVSNKALPAPIEKTISKAKGQPREPQVCTLPPSRDELTKNQVSLSCAVKGFYPSDIAVEWESNGQPENNYKTTPPVLDSDGSFFLVSKLTVDKSRWQQGNVFSCSVMHEALHNHYTQKSLSLSPGA

*Farl V_H_-C_H_1-hinge-C_H_2-C_H_3 (knobs) polypeptide:*

EVQLVESGGGVVQPGRSLRLSCSASGFTFSGYGLSWVRQAPGKGLEWVAMISSGGSYTYYADSVKGRFAISRDNAKNTLFLQMDSLRPEDTGVYFCARHGDDPAWFAYWGQGTPVTVSSASTKGPSVFPLAPSSKSTSGGTAALGCLVKDYFPEPVTVSWNSGALTSGVHTFPAVLQSSGLYSLSSVVTVPSSSLGTQTYICNVNHKPSNTKVDKRVEPKSSDKTHTCPPCPAPELLGGPSVFLFPPKPKDTLMISRTPEVTCVVVDVSHEDPEVKFNWYVDGVEVHNAKTKPREEQYASTYRVVSVLTVLHQDWLNGKEYKCKVSNKALPAPIEKTISKAKGQPREPQVYTLPPCRDELTKNQVSLWCLVKGFYPSDIAVEWESNGQPENNYKTTPPVLDSDGSFFLYSKLTVDKSRWQQGNVFSCSVMHEALHNHYTQKSLSLSPGA

*Farl V_L_-C_L_ polypeptide:*

DIQLTQSPSSLSASVGDRVTITCSVSSSISSNNLHWYQQKPGKAPKPWIYGTSNLASGVPSRFSGSGSGTDYTFTISSLQPEDIATYYCQQWSSYPYMYTFGQGTKVEIKRTVAAPSVFIFPPSDEQLKSGTASVVCLLNNFYPREAKVQWKVDNALQSGNSQESVTEQDSKDSTYSLSSTLTLSKADYEKHKVYACEVTHQGLSSPVTKSFNRGEC

*h38C2 V_H_-C_H_1-hinge-C_H_2-C_H_3 (knobs) polypeptide:*

EVQLVESGGGLVQPGGSLRLSCAASGFTFSNYWMSWVRQSPEKGLEWVSEIRLRSDNYATHYAESVKGRFTISRDNSKNTLYLQMNSLRAEDTGIYYCKTYFYSFSYWGQGTLVTVSSASTKGPSVFPLAPSSKSTSGGTAALGCLVKDYFPEPVTVSWNSGALTSGVHTFPAVLQSSGLYSLSSVVTVPSSSLGTQTYICNVNHKPSNTKVDKRVEPKSSDKTHTCPPCPAPELLGGPSVFLFPPKPKDTLMISRTPEVTCVVVDVSHEDPEVKFNWYVDGVEVHNAKTKPREEQYASTYRVVSVLTVLHQDWLNGKEYKCKVSNKALPAPIEKTISKAKGQPREPQVYTLPPCRDELTKNQVSLWCLVKGFYPSDIAVEWESNGQPENNYKTTPPVLDSDGSFFLYSKLTVDKSRWQQGNVFSCSVMHEALHNHYTQKSLSLSPGA

*(h38C2)_2_ V_H_-C_H_1-EPKSCD(G_4_S)_2_-V_H_-C_H_1-hinge-C_H_2-C_H_3 (knobs) polypeptide:*

EVQLVESGGGLVQPGGSLRLSCAASGFTFSNYWMSWVRQSPEKGLEWVSEIRLRSDNYATHYAESVKGRFTISRDNSKNTLYLQMNSLRAEDTGIYYCKTYFYSFSYWGQGTLVTVSSASTKGPSVFPLAPSSKSTSGGTAALGCLVKDYFPEPVTVSWNSGALTSGVHTFPAVLQSSGLYSLSSVVTVPSSSLGTQTYICNVNHKPSNTKVDKRVEPKSCDGGGGSGGGGSEVQLVESGGGLVQPGGSLRLSCAASGFTFSNYWMSWVRQSPEKGLEWVSEIRLRSDNYATHYAESVKGRFTISRDNSKNTLYLQMNSLRAEDTGIYYCKTYFYSFSYWGQGTLVTVSSASTKGPSVFPLAPSSKSTSGGTAALGCLVKDYFPEPVTVSWNSGALTSGVHTFPAVLQSSGLYSLSSVVTVPSSSLGTQTYICNVNHKPSNTKVDKRVEPKSSDKTHTCPPCPAPELLGGPSVFLFPPKPKDTLMISRTPEVTCVVVDVSHEDPEVKFNWYVDGVEVHNAKTKPREEQYASTYRVVSVLTVLHQDWLNGKEYKCKVSNKALPAPIEKTISKAKGQPREPQVYTLPPCRDELTKNQVSLWCLVKGFYPSDIAVEWESNGQPENNYKTTPPVLDSDGSFFLYSKLTVDKSRWQQGNVFSCSVMHEALHNHYTQKSLSLSPGA

*h38C2 V_L_-C_L_ polypeptide:*

ELQMTQSPSSLSASVGDRVTITCRSSQSLLHTYGSPYLNWYLQKPGQSPKLLIYKVSNRFSGVPSRFSGSGSGTDFTLTISSLQPEDFAVYFCSQGTHLPYTFGGGTKVEIKRTVAAPSVFIFPPSDEQLKSGTASVVCLLNNFYPREAKVQWKVDNALQSGNSQESVTEQDSKDSTYSLSSTLTLSKADYEKHKVYACEVTHQGLSSPVTKSFNRGEC

**Small molecule synthesis**

*General.* NovaSyn TG Sieber amide resin (0.21 mmol/g) was purchased from NovaBioChem. All protected amino acids and coupling reagents were purchased from Aapptec, Chem-Impex International, or NovaBioChem. All other synthetic reagents were purchased from Sigma-Aldrich, Alfa Aesar, Click Chemistry Tools, CombiBlocks, Chem-Impex International, or TCI America. The synthetic intermediates N10-(trifluoracetyl)pteroic acid (1-3) and azetidinone-OPfp (4) were synthesized as previously reported. Solid-phase peptide synthesis (SPPS) was done in 10 mL polypropylene filter columns (Thermo Scientific) using a Shaker 30 orbital shaker (SynPep Corp) at 600-800 rpm for agitation. Reverse-phase preparative HPLC was done using a Waters 2535 quaternary pump connected to a Phenomenex Gemini 10-μm C18 column (250 × 21.2 mm) with UV monitoring at 210 and 254 nm. Semi-preparative and analytical HPLC was done using an Agilent 1200 Series quaternary pump connected to either a Phenomenex Kinetix 5-μm semi-prep C18 column (250 × 10 mm) or a Phenomenex Gemini 5-μm analytical C18 column (250 × 4.6 mm) with UV monitoring at 254 nm. All HPLC methods utilized a two-solvent gradient elution with Solvent A (H_2_O + 0.1% trifluoroacetic acid (TFA)) and Solvent B (acetonitrile + 0.1% TFA). Mass spectrometry and/or LC/MS of purified products was performed on either a Shimadzu LC/MS-2020 or an Agilent G1956B LC/MSD SL system.

*Solid-phase peptides synthesis (SPPS) procedures.* Resin (typically 0.05-0.1 mmol scale) was pre-swollen in DMF (4 mL) for 1 h with shaking. The resin (either Rink or Sieber amide) was Fmoc-deprotected using 20% piperidine in DMF (4 mL) twice for 10 min each. Fmoc-protected amino acids (2-4 equivalents based on resin) were dissolved in DMF (3-4 mL) containing 4% DIEA and pre-activated by the addition of HATU (0.95 mol equivalents relative to the amino acid) for 5 min with gentle agitation. The resin was washed 4 times with DMF (6-8 mL), and the HATU-activated amino acid solution was added to the washed resin. Coupling reactions were shaken at room temperature and allowed to proceed from 3-16 h depending on the equivalents used and steric bulk of each amino acid. Coupling reactions were routinely checked for completion using the Kaiser test. Once completed, the resin was filtered and washed 4 times with DMF (6-8 mL), followed by Fmoc-deprotection using 20% piperidine in DMF (4 mL, two times, 10 min each). Cleavage from Rink resin and global deprotection was performed using 95% TFA with 2.5% triisopropylsilane (TIPS) and 2.5% H2O (4 mL, twice, 1 h each). Cleavage from Sieber resin was performed using either 33% or 5% TFA in DCM with 1% TIPS added. Some compounds require the use of Fmoc-Lys(ivDde)-OH for orthogonal protection. The ivDde group was removed using 2% hydrazine in DMF (4 mL, three times, 10 min each).

**Scheme 1.** Syntheses of β-lactam-biotin-folate **1a** and β-lactam-biotin-(folate)_2_ **2**.

*β-lactam-biotin-folate* ***1a****:*

NovaSyn TG Sieber resin (0.05 mmol scale) was reacted with the following amino acids according to the general SPPS procedure: Fmoc-Lys(ivDde), Fmoc-Lys(biotin), Fmoc-Gly, Fmoc-PEG, Fmoc-PEG, Fmoc-Glu-OtBu, and N10-(trifluoracetyl)pteroic acid. The ivDde group was removed using 2% hydrazine in DMF (4 mL, 3x 10 min each). The β-lactam conjugation moiety was installed directly on-resin by shaking with 3 equivalents of the corresponding pentafluorophenyl ester in DMF + 4% DIEA for 3 h with shaking. Cleavage and deprotection using 33% TFA in DCM + 1% TIPS followed by preparative RP-HPLC provided the completed peptide. SPPS on 0.05 mmol scale, followed by cleavage and purification by RP-HPLC (Flowrate = 10 mL/min; gradient: 10% B hold 2 min followed by gradient to 100% B over 20 min) provided **1a** (12 mg, 15% overall yield) as a pale yellow solid. LRMS (ESI+) calculated for C_71_H_99_N_19_O_21_S: 1586.7 (M+H^+^); found: 1586.5.

*β-lactam-biotin-(folate)_2_* ***2****:*

NovaSyn TG Sieber resin (0.025 mmol scale) was reacted with the following amino acids according to the general SPPS procedure: Fmoc-Lys(ivDde), Fmoc-Lys(biotin), Fmoc-Lys(Fmoc), Fmoc-PEG, Fmoc-PEG, Fmoc-Glu-OtBu, and N10-(trifluoracetyl)pteroic acid. The ivDde group was removed using 2% hydrazine in DMF (4 mL, three times, 10 min each). The β-lactam conjugation moiety was installed directly on-resin by shaking with 3 equivalents of the corresponding pentafluorophenyl ester in DMF + 4% DIEA for 3 h with shaking. SPPS on 0.025 mmol scale, followed by cleavage and purification by RP-HPLC (Flowrate = 10 mL/min, gradient: 10% B hold 2 min followed by gradient to 100% B over 20 min) provided **2** (7.6 mg, 13% overall yield) as a yellow solid. LRMS (ESI+) calculated for C_106_H_147_N_29_O_32_S: 1186.5 ([M+2H^+^]/2); found: 1186.5.

*β-lactam-biotin-LLP2A* ***3****:*

In direct adaptation of previously reported procedures (5), to pre-swelled NovaSyn TGR resin (420 mg, 0.1 mmol) was added a pre-mixed solution of Fmoc-Lys(Mtt)-OH (0.4 mmol, 4 equiv), HATU (0.4 mmol, 4 equiv), HOAt (0.1 mmol, 1 equiv) and DIEA (0.8 mmol, 8 equiv) in DMF (4 mL) and the mixture agitated at room temperature (4 h). The resin was drained, washed thoroughly with DMF and then capped with acetylimidazole and DIEA in DMF (acetylimidazole:DIEA:DMF, 1:1:8). The resin was Fmoc-deprotected using a piperidine solution (20% in DMF, 2 × 3 mL × 10 min) and washed. A pre-mixed solution of Fmoc-Orn(biotin)-OH (0.3 mmol, 3 equiv), HATU (0.3 mmol, 3 equiv), HOAt (0.1 mmol, 1 equiv) and DIEA (0.6 mmol, 6 equiv) in DMF (4 mL) was added and the mixture agitated at room temperature (overnight). The resin was capped [acetylimidazole and DIEA in DMF (acetylimidazole:DIEA:DMF, 1:1:8)], deprotected with 20% piperidine, and then agitated with a solution of Fmoc-PEG_3_-Su-OH (0.5 mmol, 5 equiv) (6), DIC (0.5 mmol, 5 equiv) and HOBt (0.5 mmol, 5 equiv) at room temperature (5 h). A second Fmoc-PEG_3_-Su-OH unit was added following similar Fmoc-deprotection and coupling steps and the LLP2A peptide was installed using the method and reagents previously described (7). The resin was cleaved (TFA:TFA:TIS:H_2_O, 95:2.5:2.5) and crude product was precipitated from Et_2_O and purified by reverse-phase HPLC to afford peptide **14** (16.6 mg, 10% yield). LRMS *m/z*: 871 (MH_2_^2+^), 571 (MH_3_^3+^). To a stirred solution of peptide **14** (5.7 mg, 6.03 μmol) in DMF (24 μL) were added DMF solutions of DIEA (0.5M, 50 μL, 4 equiv) and DBCO-Su-OSu (0.5M, 18 μL, 1.5 equiv), respectively and the mixture was stirred at room temperature (20 min). A DMF solution of azide **13** (0.5 M, 25 μL, 2 equiv) was added and the mixture was stirred at room temperature (1 h), then the mixture was purified directly by reverse-phase HPLC to afford **3** (2.4 mg, 47%). HRMS: calculated (MH_3_^+3^) 790.7233, observed (MH_3_^+3^) 790.7210; Δ = 2.3 ppm.

**Scheme 2.** Synthesis of β-lactam-biotin-LLP2A **3** via peptide **14**.

*Synthesis of building blocks***:**

Biotin-pentafluorophenyl ester **4**. Using a modified literature procedure (8), to a solution of biotin (5.0 g, 20.5 mmol) in DMF (68 mL) (gently heating) was added in one portion bis-pentafluorophenylcarbonate (8.87 g, 22.5 mmol, 1.1 equiv). Triethylamine (3.28 mL, 23.5 mmol, 1.15 equiv) was added drop-wise via syringe and the resulting solution was brought to 60 °C and stirred (2 h), then cooled to room temperature and diluted with two volumes of Et_2_O. The resulting white precipitate was collected by filtration and the filtrate was concentrated *in vacuo* to an approximated volume of 35 mL and again diluted with two volumes of Et_2_O. The resulting white precipitate was collected by filtration and combined with the first crop to afford Pfp-ester **4** (7.3 g, 87% yield). Spectral data consistent with literature values (8). ^1^H NMR (500MHz, DMSO-d_6_) δ 6.48 - 6.32 (m, 2H), 4.34 - 4.28 (m, 1H), 4.18 - 4.12 (m, 1H), 3.12 (ddd, *J*=8.3, 6.3, 4.4 Hz, 1H), 2.87 - 2.76 (m, 3H), 2.59 (d, *J*=12.7 Hz, 1H), 1.75 - 1.61 (m, 3H), 1.57 - 1.37 (m, 3H). LRMS *m/z*: 411 (M+H), 409 (M-H).

**Scheme 3.** Synthesis of compound **4**.

Pentafluorophenyl pent-4-ynoate **5**. A stirred mixture of pentynoic acid (1.0 g, 10.2 mmol), bis-pentafluorophenyl carbonate (4.42 g, 11.22 mmol, 1.1 equiv) and triethylamine (1.563 mL, 11.22 mmol, 1.1 equiv) in THF (20.5 mL) was brought to 60 °C (1 h) and then stirred at room temperature (overnight). The reaction mixture was concentrated *in vacuo* and triturated with Et_2_O:hexanes (1:1) and the resulting precipitate was collected by filtration to afford **5** as a white solid (2.29 g, 85% yield). Analytical data was consistent with reported values (9). ^1^H NMR (400MHz, CHLOROFORM-d) δ 2.96 - 2.91 (m, 2H), 2.66 (td, *J*=7.3, 2.5 Hz, 2H), 2.06 (t, *J*=2.7 Hz, 1H). LRMS *m/z*: 265 (M+H).


**Scheme 4.** Synthesis of compound **5**.

*N*-(4-(3-Oxo-3-(2-oxoazetidin-1-yl)propyl)phenyl)pent-4-ynamide **8**. To a stirred solution of 1-(3-(4-nitrophenyl)propanoyl)azetidin-2-one (10) (**6**, 500 mg, 2.014 mmol) in MeOH (20 mL) at 0 °C under argon, was added acetyl chloride (1.432 mL, 20.14 mmol, 10 equiv) drop-wise via syringe. To this solution was added 10% Pd•C (214 mg, 0.2 mmol, 0.1 equiv) and the resulting slurry was stirred vigorously under a H_2_ atmosphere (1.5 h). The reaction mixture was filtered through Celite and the filtrate was concentrated *in vacuo* and diluted with a solution of 1 N HCl in Et_2_O. The resulting precipitate was collected by filtration to afford azetidinone **7** as a white solid (517 mg, quantitative). To this crude **7** and **5** (1.062 g, 4.02 mmol, 2 equiv) in DMF (6.5 mL) was added triethylamine slowly via syringe and the resulting mixture was stirred at room temperature (overnight). The reaction mixture was concentrated *in vacuo* and purified by silica gel flash chromatography to afford **8** as an off-white solid (431 mg, 72% yield). ^1^H NMR (500MHz, CHLOROFORM-d) δ 7.70 (s, 1H), 7.42 (d, *J*=8.3 Hz, 2H), 7.16 (d, *J*=8.3 Hz, 2H), 3.67 (dt, *J*=13.3, 6.8 Hz, 2H), 3.56 (t, *J*=5.4 Hz, 2H), 3.15 (q, *J*=7.3 Hz, 2H), 3.06 - 3.01 (m, 2H), 3.00 - 2.96 (m, 2H), 2.95 - 2.91 (m, 2H), 2.04 (s, 1H). LRMS *m/z*: 299 (M+H).


**Scheme 5.** Synthesis of compound **8**.

Fmoc-Orn(biotin)-OH **10.** To a cooled solution of Fmoc-Orn(HCl)-OH (1.905 g, 4.87 mmol) and triethylamine (1.698 ml, 12.18 mmol) in DMF (32.5 mL) was added a solution of biotin-Pfp ester (**4**, 2.0 g, 4.87 mmol) in DMF (16.25 mL) drop-wise via pipette and the mixture was stirred at room temperature (1.5 h). The mixture was concentrated *in vacuo* and the residue was added to stirring Et_2_O to provide a white precipitate, which was collected by filtration and dried *in vacuo* to afford **10** (2.82 g, 99% yield). ^1^H NMR (400MHz, DMSO-d6) δ 7.89 (d, J=7.5 Hz, 2H), 7.80 (br. s., 1H), 7.72 (d, J=7.3 Hz, 2H), 7.47 - 7.37 (m, 3H), 7.36 - 7.29 (m, 2H), 6.45 - 6.33 (m, 2H), 4.33 - 4.19 (m, 4H), 4.12 (dd, J=4.5, 1.7 Hz, 1H), 3.91 - 3.84 (m, 1H), 3.08 (dd, J=4.4, 2.4 Hz, 1H), 3.02 (d, J=6.2 Hz, 2H), 2.80 (dd, J=12.4, 5.1 Hz, 1H), 2.57 (d, J=12.4 Hz, 1H), 2.05 (t, J=7.4 Hz, 2H), 1.76 - 1.67 (m, 1H), 1.64 - 1.40 (m, 8H), 1.35 - 1.26 (m, 2H). LRMS *m/z*: 581 (M+H), 579 (M-H).

**Scheme 6.** Synthesis of compound **10**.

5-Azidopentanoyl chloride **11**. The following method has been previously reported without experimental detail (11). To a solution of 5-azidopentanoic acid (500 mg, 3.49 mmol) in THF (8.7 mL), at room temperature under argon, was added SOCl_2_ (0.382 mL, 5.24 mmol, 1.5 equiv) drop-wise via syringe, followed by 1 drop of DMF. The mixture was stirred at room temperature (overnight) and then concentrated *in vacuo* to afford acid chloride **11** (558 mg, 99% yield). ^1^H NMR (400MHz, CDCl_3_) δ 3.37 - 3.28 (m, 2H), 2.95 (t, J=7.1 Hz, 2H), 1.88 - 1.74 (m, 2H), 1.73 - 1.59 (m, 2H). [Note: Prepare immediately prior to use and store at low temperature as a 1 M solution in THF.]


**Scheme 7.** Synthesis of compound **11**.

5-Azido-*N*-(4-(3-oxo-3-(2-oxoazetidin-1-yl)propyl)phenyl)pentanamide **13.** A slurry of azetidinone **6** (200 mg, 0.806 mmol) and 10% Pd•C (171 mg, 0.161 mmol, 0.2 equiv) in THF (4.028 mL), was stirred vigorously under H2 at 0 °C and monitored closely by TLC. Upon full consumption of starting material, the reaction mixture was filtered through Celite directly into a cooled (0 °C) solution of azido acid chloride **11** (2.417 mL, 2.417 mmol, 3 equiv) under argon and 2,6-di-*tert*-butylpyridine (0.905 mL, 4.03 mmol, 5 equiv) was added via syringe and the mixture was stirred at room temperature (overnight). The reaction mixture was filtered, concentrated *in vacuo* and directly purified by silica gel flash chromatography to afford **13** (255 mg, 92% yield). ^1^H NMR (400MHz, CDCl_3_) δ 7.47 (br. s., 1H), 7.41 (d, *J*=7.9 Hz, 2H), 7.17 (d, *J*=7.8 Hz, 2H), 3.55 (t, *J*=5.1 Hz, 2H), 3.31 (t, *J*=6.4 Hz, 2H), 3.06 - 2.88 (m, 6H), 2.37 (t, *J*=7.0 Hz, 2H), 1.85 - 1.74 (m, 2H), 1.72 - 1.61 (m, 2H). LRMS *m/z*: 344 (M+H), 366 (M+Na), 342 (M-H).

**Scheme 8.** Synthesis of compound **13**.

**SUPPLEMENTARY REFERENCES**

1. Godwin HA, Rosenberg IH, Ferenz CR, Jacobs PM, Meienhofer J. The synthesis of biologically active pteroyloligo- 0L-glutamates (folic acid conjugates). Evaluation of ( 3 H)pteroylheptaglutamate for metabolic studies. *J Biol Chem* (1972) 247:2266-71.

2. Harvison PJ, Kalman TI. Synthesis and biological activity of novel folic acid analogues: pteroyl-S-alkylhomocysteine sulfoximines. *J Med Chem* (1992) 35:1227-33.

3. Luo J, Smith MD, Lantrip DA, Wang S, Fuchs PL. Efficient syntheses of pyrofolic acid and pteroyl azide, reagents for the production of carboxyl-differentiated derivatives of folic acid. *J Am Chem Soc* (1997) 119:10004-13.

4. Magano J, Bock B, Brennan J, Farrand D, Lovdahl M, Maloney MT*,* et al. Chromatography- and lyophilization-free synthesis of a peptide-linker conjugate. *Org Process Res Dev* (2014) 18:142-51.

5. Thomas JD, Hofer T, Rader C, and Burke TR Jr. Application of a trifunctional reactive linker for the construction of antibody–drug hybrid conjugates. *Bioorg Med Chem Lett* (2008) 18:5785-8.

6. Song A, Wang X, Zhang J, Mařı́k J, Lebrilla CB, and Lam KS. Synthesis of hydrophilic and flexible linkers for peptide derivatization in solid phase. *Bioorg Med Chem Lett* (2004) 14:161-5.

7. Peng L, Liu R, Marik J, Wang X, Takada Y, and Lam KS. Combinatorial chemistry identifies high-affinity peptidomimetics against alpha4beta1 integrin for in vivo tumor imaging. *Nat Chem Biol* (2006) 2:381-9.

8. Kessler D, Roth PJ, and Theato P. Reactive surface coatings based on polysilsesquioxanes: controlled functionalization for specific protein immobilization. *Langmuir* (2009) 25:10068-76.

9. Fiehn T, Goddard R, Seidel RW, and Kubik S. A cyclopeptide-derived molecular cage for sulfate ions that closes with a click. *Chem Eur J* (2010) 16:7241-55.

10. Palanki MS, Bhat A, Lappe RW, Liu B, Oates B, Rizzo J, Stankovic N, and Bradshaw C. Development of novel linkers to conjugate pharmacophores to a carrier antibody. *Bioorg Med Chem Lett* (2012) 22:4249-53.

11. Chaturvedi D, Chaturvedi AK, Mishra N, and Mishra V (2012) An efficient and novel approach for the synthesis of substituted N-aryl lactams. *Org Biomol Chem* (2012) 10:9148-51.

**SUPPLEMENTARY FIGURES AND FIGURE LEGENDS**

**FIGURE S1.** Analysis of purified biAbs. (**A**) SDS-PAGE and Coomassie Blue staining analysis of purified v9 × Farl showing the expected bands at ~125 kDa under nonreducing (nr) and ~50 kDa and ~25 kDa under reducing (r) conditions (upper panel). SEC analysis of v9 × Farl eluting as major peak at 13.03 mL (bottom panel). (**B**) Corresponding analyses of purified v9 × h38C2 showing the expected bands at ~125 kDa under nonreducing (nr) and ~50 kDa and ~25 kDa under reducing (r) conditions (upper panel) and elution as major peak at 13.17 mL (bottom panel). (**C**) Corresponding analyses of purified v9 × (h38C2)_2_ showing the expected bands at ~175 kDa under nonreducing (nr) and ~75 kDa, ~50 kDa and ~25 kDa under reducing (r) conditions (upper panel) and elution as major peak at 12.26 mL (bottom panel).

**FIGURE S2.** Cell surface binding of conventional FOLR1-targeting biAb. (**A**) Flow cytometry analysis of conventional biAb v9 × Farl binding to CD3+ FOLR1− human T-cell line Jurkat and FOLR1+ CD3− human ovarian cancer cell line IGROV-1 using 20 nM biAbs followed by Alexa Fluor 647-conjugated goat anti-human IgG-Fc pAbs. (**B**) Titration curve of conventional biAb v9 × Farl binding to IGROV-1 cells detected with Alexa Fluor 647-conjugated goat anti-human IgG pAbs (left panel). Saturation analysis of v9 × Farl binding to IGROV-1 cells (right panel). Shown are mean ± SD values from independent triplicates.

**FIGURE S3.** Synthetic compounds for chemical programming. Structures of monovalent compounds β-lactam-biotin-folate **1a** and **1b**, bivalent compound β-lactam-biotin-(folate)_2_ **2**, and integrin α_4_β_1_-targeting compound β-lactam-biotin-LLP2A **3**.


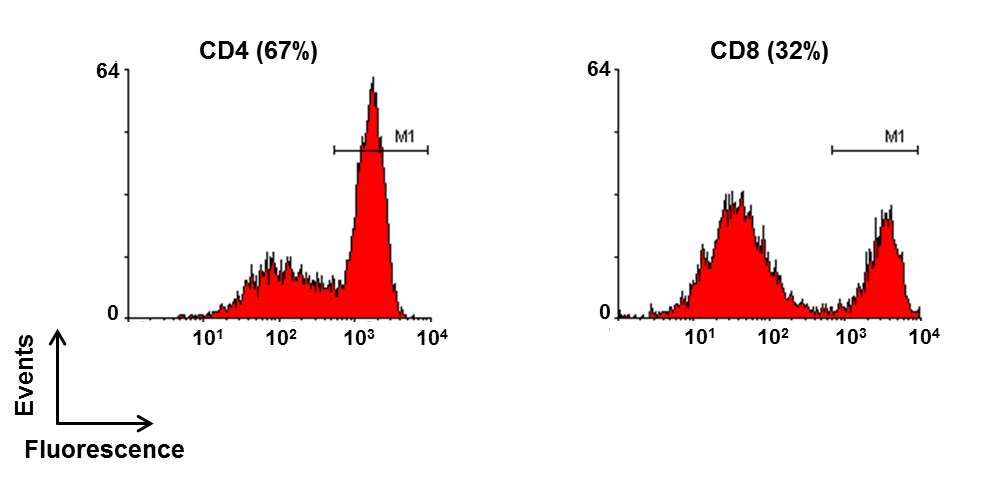


**FIGURE S4.** Flow cytometry analysis of *ex vivo* expanded T cells. The expanded T cells were stained with 2 µg/mL mouse anti-human CD4 (left) or CD8 (right) followed by APC-conjugated goat anti-mouse IgG pAbs.


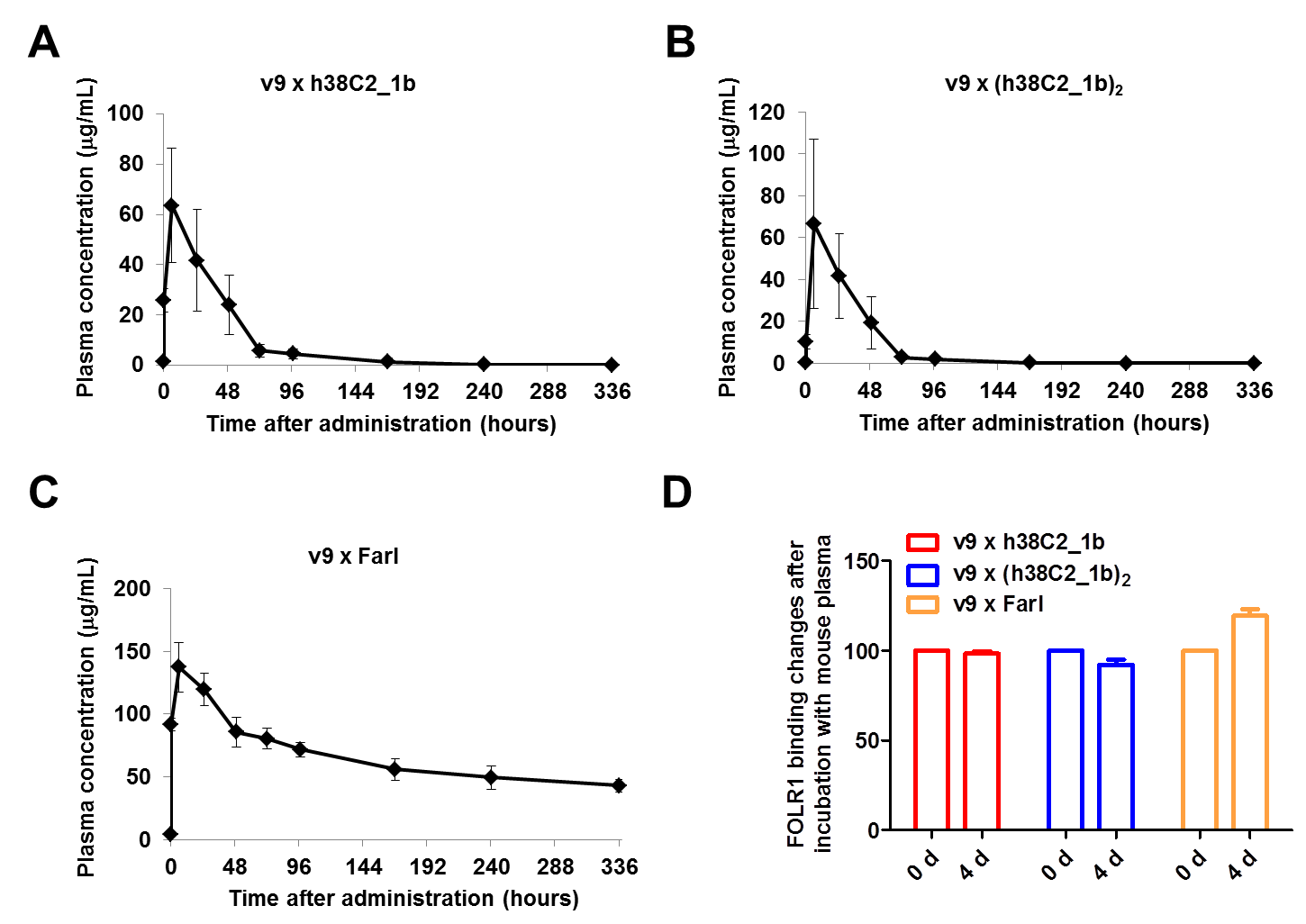


**FIGURE S5.** PK and stability of FOLR1-targeting biAbs. Four female CD-1 mice were injected i.p. with 6 mg/kg v9 × h38C2_**1b** (**A**), v9 × (h38C2_**1b**)_2_ (**B**) and v9 × Farl (**C**). The plasma concentrations of the biAbs at the indicated time points were quantified with flow cytometry, using Alexa Fluor 647-conjugated goat anti-human IgG Fc-specific pAb for detection of biAbs binding to IGROV-1 cells. Shown are mean ± SD values for each time point. PK parameters are listed in **Table 2**. (**D**) FOLR1 binding of the indicated three biAbs before and after 4-day incubation with mouse plasma at 37 °C was determined by flow cytometry using IGROV-1 cells and normalized (0 d ≡ 100%). Shown are mean ± SD values.


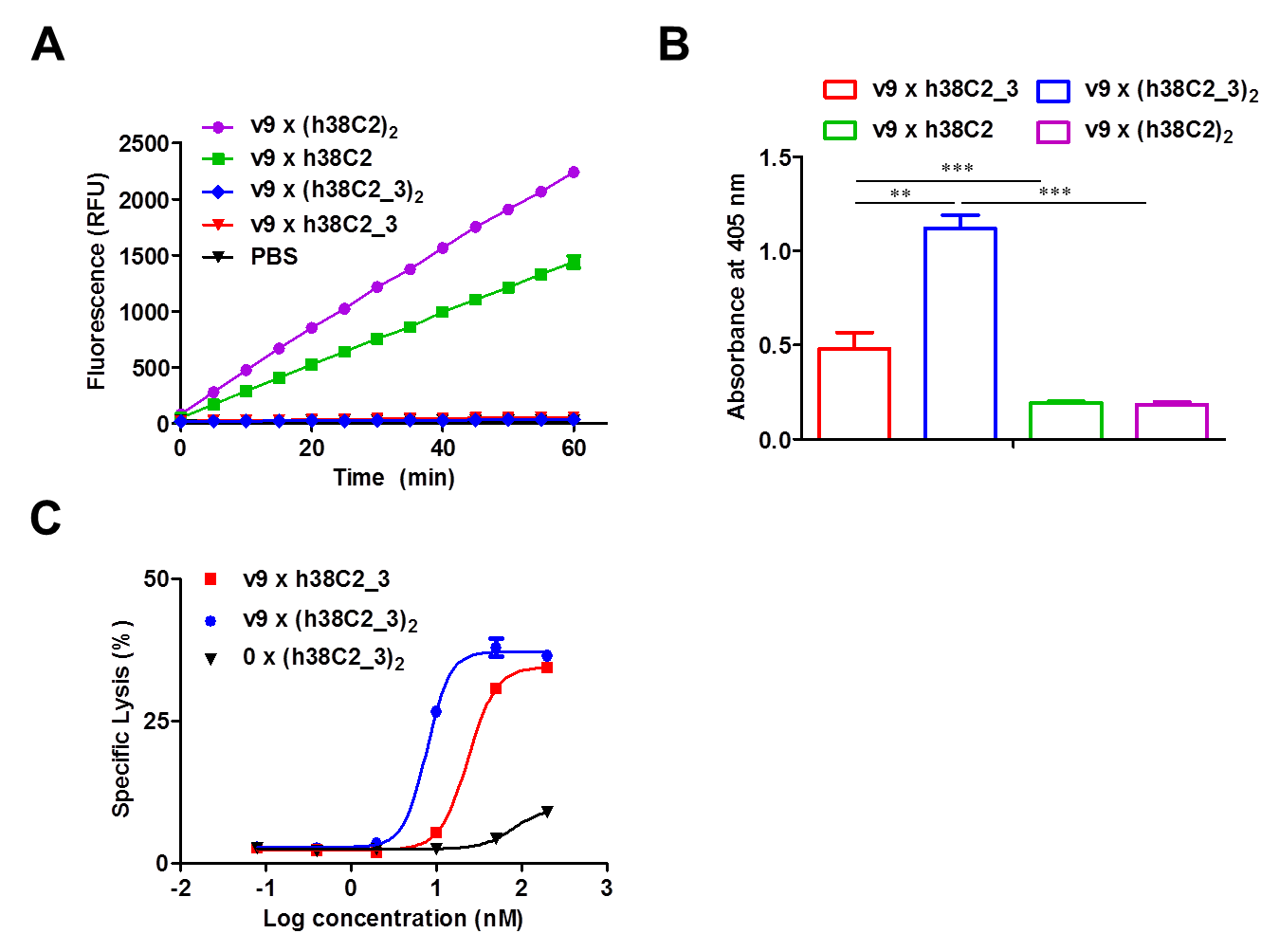


**FIGURE S6.** Characterization of chemically programmed integrin α_4_β_1_-targeting biAbs. (**A**) Catalytic retro-aldol activity of unprogrammed and chemically programmed h38C2-containing biAbs. The signal is reported in relative fluorescent units (RFU; mean ± SD of triplicates). PBS was used as negative control. (**B**) Compound **3**-programmed h38C2-containing biAbs binding to immobilized human integrin α_4_β_1_ in the presence of 1 mM MnCl_2_ as detected with HRP-conjugated goat anti-human IgG pAbs. Shown are mean ± SD values from independent triplicates. An unpaired two-tailed t-test was used to analyze significant differences (**, p < 0.01; ***, p < 0.001). (**C**) Cytotoxicity of biAbs tested with *ex vivo* expanded primary human T cells (effector cells) and JeKo-1 cells (target cells) at an effector-to-target cell ratio of 10:1 and measured after 16-h incubation. Shown are mean ± SD values from independent triplicates.
